# The person-based development and realist evaluation of a pre-consultation form for GP consultations

**DOI:** 10.3310/nihropenres.13249.1

**Published:** 2022-02-17

**Authors:** Mairead Murphy, Chris Salisbury, Anne Scott, Lucia Sollazzi-Davies, Geoff Wong

**Affiliations:** 1 Bristol Medical School, University of Bristol, Bristol, BS8 2PS, UK; 2 West Walk GP Practice, Bristol, UK; 3 Nuffield Department of Primary Care Health Sciences, University of Oxford, Oxford, OX2 6HT, UK

**Keywords:** e-PROs, GP consultations, GP-patient communication, person-based approach, realist evaluation

## Abstract

**Background:**

Use of telephone, video and e-consultations is increasing. These can make consultations more transactional, potentially missing patients’ concerns. This study aimed to develop a complex intervention to address patients’ concerns more comprehensively in general practice and test the feasibility of this in a cluster-randomised framework.

The complex intervention used two technologies: a patient-completed pre-consultation form used at consultation opening and a doctor-provided summary report provided at consultation closure. This paper reports on the development and realist evaluation of the pre-consultation questionnaire.

**Methods:**

A person-based approach was used to develop the pre-consultation form. An online questionnaire system was designed to allow patient self-completion of a form which could be shared with GPs. This was tested with 45 patients in three rounds, with iterative adjustments made based on feedback after each round.

Subsequently, an intervention incorporating the pre-consultation form with the summary report was then tested in a cluster-randomised framework with 30 patients per practice in six practices: four randomised to intervention, and two to control. An embedded realist evaluation was carried out. The main feasibility study results are reported elsewhere.

**Results:**

Intervention Development: 15 patients were recruited per practice. Twelve patients, six GPs and three administrators were interviewed and 32 changes were made iteratively in three rounds. Recruitment rates (proportion of patients responding to the text) increased from 15% in round one to 50% in round three.

Realist evaluation: The pre-consultation form was most useful for people comfortable with technology and with hidden concerns or anxiety about the consultation. It resulted in more issues being discussed and support provided, more effective use of time and greater patient satisfaction.

**Conclusions:**

The person-based approach was successful. The pre-consultation form uncovers more depth and improves satisfaction in certain consultations and patients. Technological improvements are required before this could be rolled out more widely.

## Plain English summary

The problem: For some patients, GP consultations are too short. Sometimes patients’ problems are missed. We wanted to improve GP consultations.

What we did: We developed a better way to start and end consultations using a new digital method. Before a GP consultation, patients fill in a form online that lets them describe their problems. This is shared with their GP. At the end of the consultation GPs can give patients a one-page summary of what was discussed. This paper reports on how we developed and tested the online pre-consultation form.

How we tested it: We first piloted the online form in three GP practices in turn. We interviewed patients, GPs and an administrator in each practice and made changes based on their suggestions. Each new version was tested in a new practice.

We then tested the final online form together with the one-page summary in four practices with 30 patients each. We interviewed patients and GPs to find who it was most useful for and when.

What we found: GPs and patients agreed the final version of the online form was much better than the first version. The percentage of patients who filled in a form after getting a text message increased from 15% in practice 1 to 50% in practice 3.

By testing the final form in four practices, we found it worked best for people who find technology easy to use and have hidden concerns or worries about the consultation. These patients found their GP was better prepared and problems were dealt with better. GPs thought consultations were more efficient.

Conclusions: The approach we took was very successful in developing the online form and patients and GPs found it useful. The technology would need to be improved before it could be rolled out more widely.

## 1 Background

### 1.1 Rationale for study

Patients often leave GP consultations with unaddressed concerns ^1, 2^. This can lead to high rates of re-consultation and increased morbidity in the population. Previous research shows that approximately 27% of patients consulting in primary care have seen a doctor or nurse for the same problem in the last four weeks ^3^, and more recently published research demonstrates that up to 50% of consultations in primary care are followed by another consultation within two weeks ^4^. Although there are no estimates of re-consultation for unaddressed concerns in primary care, we know that problems are missed in up to 50% of primary care consultations ^2^, and that reducing consultation rates by just 1% in 2016 could have saved the NHS over £100 million ^5^. Primary care patients often present with multiple complex problems, many of which are unrelated to physical symptoms, and include informational needs on symptom-management or self-care, emotional problems, health concerns or social problems ^6^. In the context of multiple presenting problems, GPs tend to focus on physical symptoms ^7^. While this prioritisation is entirely appropriate to ensure correct diagnosis and patient safety, any missed opportunities to improve patient understanding and ability to self-care are also costly: a study in 2015 found that increasing patient engagement in their own health could save the NHS £2 billion by 2020 ^8^. Small changes to improve the ability of GPs to thoroughly and efficiently address patients’ presenting problems, concerns and questions could therefore have considerable impact on the overall NHS budget as well as on patient and doctor well-being and satisfaction.

The Calgary-Cambridge guide, which is used as a basis for training medical students and doctors, identifies six steps to conducting a GP consultation: initiating, information gathering, providing structure, relationship building, explanation/planning and closing ^2^. Opportunities to address patients problems are commonly missed at consultation initiation (when the GP should elicit the patients reason for attendance) ^2^. Problems can remain unaddressed at consultation closure, if advice given is unclear, particularly with regards “safety-netting”: i.e. advising patients what to do if the problem does not resolve, or gets worse ^9^. Research suggests that interventions at each end of the consultation can help to address patient concerns. At consultation initiation, sharing the results from patient-reported outcome measures (PROMs) with clinicians can help to elicit concerns ^10^. At consultation closure, providing the patient with written information as well as spoken can improve recall and adherence ^11^.

To help with this problem, we designed an intervention: the Consultation Open and Close (COAC) intervention which used a patient-filled pre-consultation form, completed prior to and discussed at consultation opening, and a summary report provided by the doctor at consultation closure. We then tested these interventions in a feasibility study.

### 1.2 Review of Evidence of pre-consultation forms

***1.2.1 Consultation initiation: eliciting all concerns***

Active listening was described by Carl Rogers as absorbing everything a person says without “subtracting” or “amending” ^12^. Many patients regard the ability to listen as the single most important characteristic of a good doctor ^13^. The importance of active listening has long been recognised and incorporated into undergraduate medical curriculae ^14^.

Despite this, studies have shown that GPs often interrupt patients, particularly during the patient’s opening statement (or patient monologue) ^2^. Although GPs may perceive that the patient monologue is wasting time, in fact, it takes only 30 seconds on average ^15^. One study showed that doctors waited an average of 23 seconds before interrupting the patient’s opening statement ^1^, when less than ten seconds more would usually allow the patient to finish. When GPs interrupt, they are nearly always doing so with their patients interests in mind: recognising the importance of listening, but having limited time to gather essential information from patients before moving onto diagnosis and advice ^16^. In many cases, GPs interrupt because a patient is providing medical history which the clinician already knows. One approach to dealing with this problem is “physician goes first”: whereby the doctor starts the consultation with a very short synopsis of what he/she knows about the patient’s recent medical history, before asking the patient about their goals for consulting and allowing them to speak uninterrupted ^17^.

This approach can be facilitated by a review of the patient’s medical record before the start of the consultation, and also by patient completion of a PROM, which is shared with the clinician before the consultation. This can save valuable consultation time, by giving the GP or nurse an immediate oversight of the patient’s current state of health and immediate presenting problems ^18^.

***1.2.2 Use of electronic PROMs in primary care at an individual level***

PROMs were originally designed for use at aggregate level, to compare the scores of groups of patients receiving different care ^10^. However, PROMs are increasingly being used at an individual-level to inform a consultation, set priorities or aid diagnosis ^10^. Feedback of individual-level PROMs information to clinicians has been used most widely in oncology ^19^. It has a positive effect on patient experience and patient care by promoting patient self-reflection thereby helping patients remember their main concerns ^20^, by improving patient-clinician communication ^21^ and by making it easier for patients to share information which they find it difficult to express verbally ^22^. There is less evidence for an impact on outcomes ^19^. Trials of PROMs feedback to clinicians which *have* shown effects on patient outcome tend to use randomisation at the physician or practice level, rather than the patient-level, i.e. with each practice randomly assigned to using PROMs feedback rather than each patient ^23^.

A realist review of feedback of individual-level PROMs to clinicians found that one mechanism by which they can work is by raising clinicians’ awareness of patient concerns ^10^. In the context of increasing GP workload, it is important that these PROMs capture relevant information, delivered succinctly. Benefits of electronic PROMs (ePROMs) include; remote completion, instant transfer, and filtering and summarising of data so clinicians see only the most important information. They also solve problems with questionnaire completion in waiting rooms; most primary care patients book an appointment only one or two days in advance so recruiting patients before a consultation normally requires waiting room recruitment ^24^. This limits the time for form completion, and some patients will be called in to their consultation before completing the questionnaire ^25^. The current widespread digitisation in general practice ^26^ offers a timely opportunity to integrate an ePROM into clinical practice for use at an individual-level to help identify patient concerns.

Electronic triage forms were mandated by the NHS long term plan ^27^ and were rolled out across general practice during this study. Electronic triage forms have features that are common to ePROMs completed before consultations; they both collect clinical information from the patient which is shared asynchronously with a clinician. However, they differ in purpose and content. Electronic triage forms are primarily used as a triage tool and collect information on symptoms. The patient may not receive a consultation after completion of an electronic triage form, but can be advised to self-care, go to a pharmacist or Emergency Department (ED) or receive advice from the GP through email or the triage portal. The primary purpose of ePROMs shared with clinicians before consultations is not triage, as the patient has already has a booked appointment; it can serve multiple purposes, include uncovering additional information or providing more detail on a patient’s problems ^10^.

The aim of this study was to develop and test an intervention to more comprehensively address patients’ concerns in general practice through use of a pre-consultation form discussed at consultation opening and provision of a summary report on consultation closure.

This paper describes the development of the pre-consultation form and a realist evaluation of its use from the feasibility study of the COAC intervention. The development and testing of the consultation summary report and the full feasibility study results are reported separately in two papers published alongside this one.

## 2 Methods

### 2.1 Study setting

This study was based in primary care involving general practices serving different patient populations in Bristol, North Somerset and South Gloucestershire (BNSSG). Practices were selected from areas within a range of socioeconomic deprivation levels as well as urban, suburban and rural areas.

The COVID-19 pandemic occurred six months into this study. Under an NIHR directive, the study was paused in March 2020 and restarted in September 2020. Research protocols were updated so the intervention and research did not require face-to-face contact. 

### 2.2 The Consultation Open and Close Study

The COAC Study involved the development and testing of an intervention, incorporating use of an individual-level PROM at consultation opening and written information at consultation closure. The primary aim of the COAC study was to develop and test the feasibility of a complex intervention designed to more comprehensively address patients’ concerns in general practice, thereby reducing re-consultation rates, improving patients’ well-being and health knowledge, reducing health concerns and increasing patients’ confidence in their health provision and health plan.

The COAC study incorporated two phases: an Intervention Development study (Study 1) and a feasibility study (Study 2) as follows:

***Intervention development study:*** This involved design of a complex intervention to improve the ability of GPs or nurse practitioners to address patients’ concerns through a) development and testing an electronic patient questionnaire at consultation opening and b) developing and testing a summary report at consultation closure, which is either printed or texted to the patient or is accessible from the patient record. These were designed and evaluated separately, in accordance with MRC guidance for design of complex interventions ^28^.

***Feasibility study:*** In this study, the COAC intervention was tested in a cluster-randomised framework to establish the feasibility of both a randomised-control trial of the intervention and the intervention itself.

**Figure 1.**
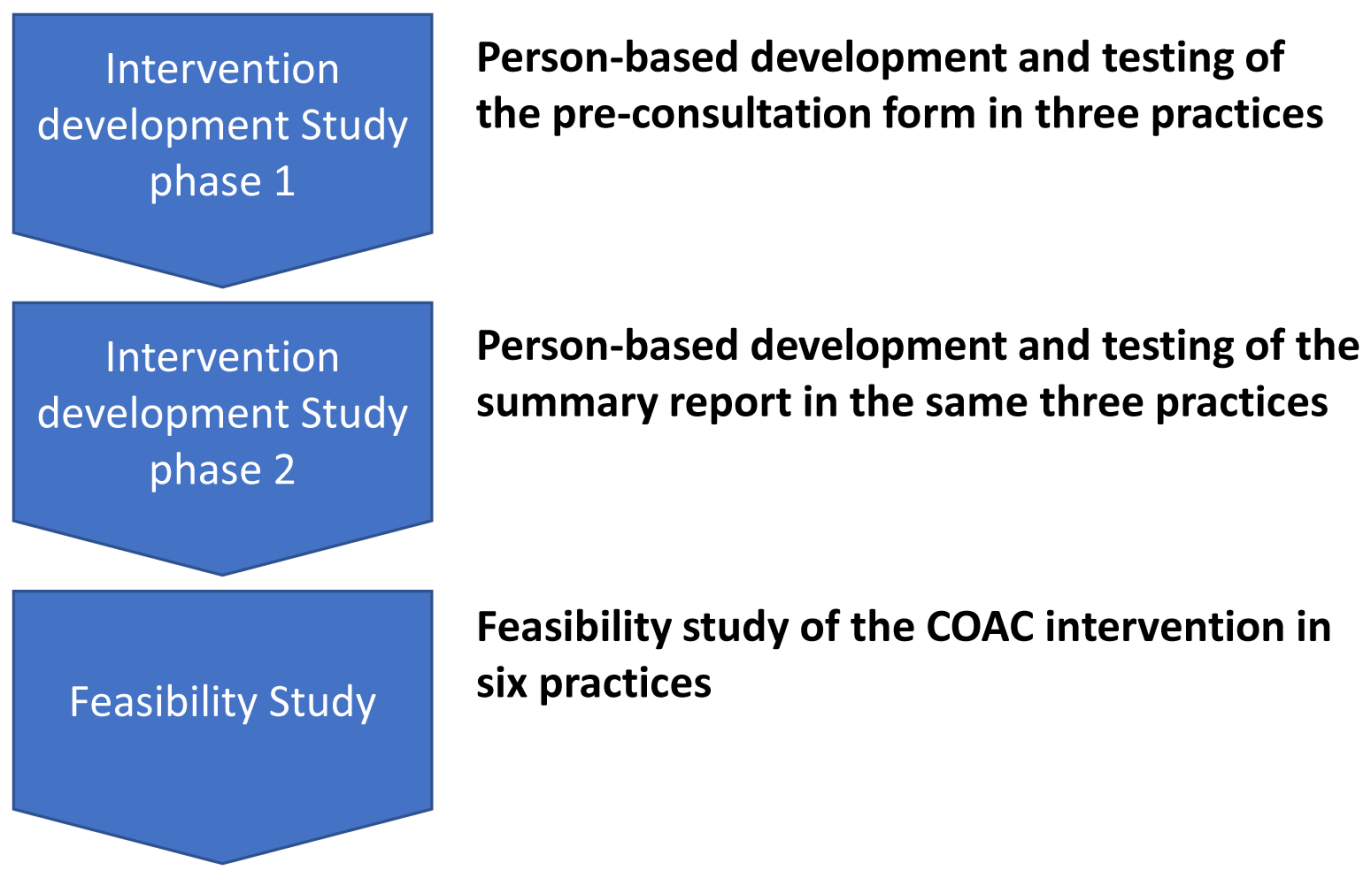
The Intervention Development and Feasibility Studies.

The sequential nature of the studies is shown in Figure 1.

This paper describes the development of the pre-consultation form and a realist evaluation of its use from the feasibility study of the COAC intervention. The development and testing of the consultation summary report and the full feasibility study results are reported separately in two papers published alongside this one. 

### 2.3 Recruitment

***2.3.1 Practice recruitment***

Practices were approached by the NIHR Clinical Research Network for the West of England (hereafter referred to as the CRN) with the information on the study. Practices were recruited to the two phases separately; with practices who participated in the Intervention Development study actively encouraged to continue their participation in the Feasibility study.

For each study, the CRN shared the study Research Information Sheet for Practices (RISP) with practices who met the inclusion criteria. Interested practices then contacted the study chief investigator (CI) who arranged a meeting(s) with the practice manager, or GP research lead.

Practice representatives were asked to sign a practice agreement consenting to the practice taking part in the study. Practices were approached for Study 1 in November 2019 (three practices were required for Study 1); and for Study 2 in May 2021 (six practices were required for Study 2).

All selected practices already used SMS software (MJOG and accuRx) and the patient record system EMIS. Administrators were expected to be familiar with the process of sending batch texts using practice SMS software (e.g. MJOG) and in uploading reports to and setting alerts in EMIS.

***2.3.2 Eligibility criteria***

For the Intervention Development study (three practices) we purposively selected: one practice in the top deprivation quartile, one at the median, and one in the lower quartile. For the feasibility study (six practices), we selected three practices in the top two deprivation quartiles and three practices in the bottom two.

Patients in both studies were included who were:

▪      Aged 17 or over (on date of SMS invitation to participate)

▪      Had an upcoming appointment with a recruiting GP within the next week.

Patients were excluded if they were:

▪      Housebound

▪      Had not given permission to receive SMS messages from the practice

▪      Had a recent diagnosis of life-limiting or life-threatening illness

▪      Were deemed by the GP to be at serious suicidal risk

▪      Were unable to complete questionnaires in English even with the help of carers.

The remainder of the methods are described separately for the Intervention Development study and feasibility study in the respective two sections that follow.

### 2.4. Intervention development study methods

***2.4.1 Approach***

**Figure 2.**
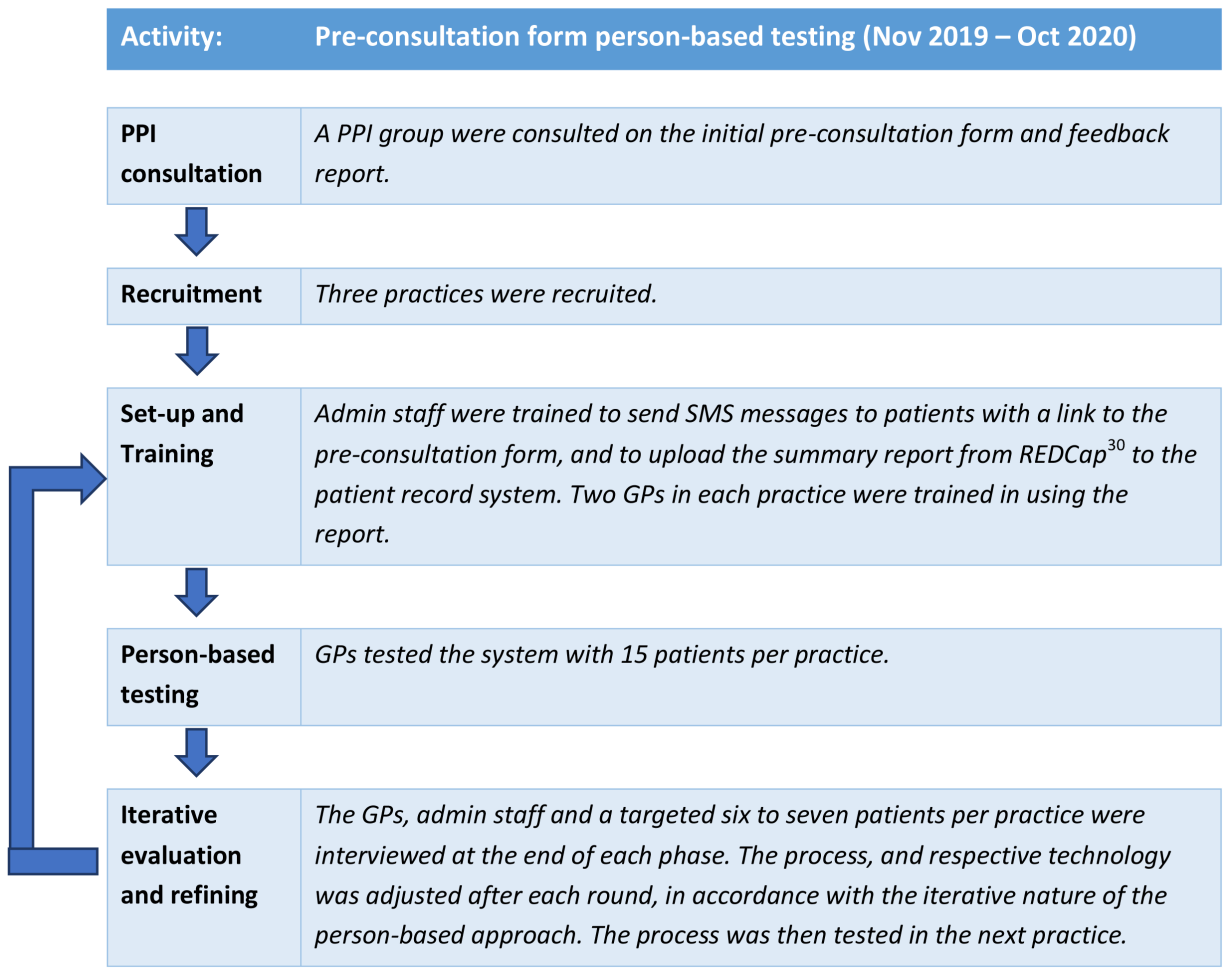
Person-based approach taken to develop the pre-consultation form.

The Intervention Development study was carried out in two distinct parts, one for development of the online pre-consultation form and one for development of the summary report provided at consultation closure. Development of the pre-consultation form is described in this section. A prototype was developed based on the research literature and a series of patient and public involvement (PPI) group consultations. This was then tested with actual patients using a person-based approach, which involves using mixed-methods research to systematically investigate the needs, attitudes and situation of the people who will be using the intervention ^29^. Through the person-based approach, each step of the intervention was tested in rounds and adjusted after each round according to the feedback given from patients and clinicians. This iterative approach is shown in Figure 2.

***2.4.2 Prototype development***

**Starting position**

Pilot work with a PPI group suggested that the pre-consultation form should include both individualised information (a list, generated by the patient, of their reasons for attending, and the key issues they would like to discuss) and standardised information (a short list of questions on common problems, with tick-box answers). A standard questionnaire and report were developed before the study commenced, based on the Primary Care Outcomes Questionnaire (PCOQ) and this was used as the starting point for person-based development and testing. The PCOQ is a validated generic questionnaire which was developed to capture the main outcomes which can be influenced by primary care. It has 24 items which include physical and emotional symptoms and function, self-care, health behaviour, adherence, and a sense of support ^25, 30^.

The pre-consultation form was put into an online survey using the University of Bristol database system REDCap: a low-cost, secure, web-based electronic data capture system for clinical research ^31^. Only 18 of the 24 PCOQ items were included, as six items refer to the patient’s confidence in seeking healthcare, and are not suitable for sharing with their clinician. Versions were developed for smartphone and computer.

A process was designed for the information from the pre-consultation form to be downloaded from REDCap and attached to EMIS in a pdf report format for the GP or nurse to review before the consultation. Rather than simply attaching the full questionnaire, this was formatted so that it was short and easy for clinicians to digest. It contained two sections: an individualised section with the patients’ reasons for attending, and a standardised section, which was a colour-coded list of responses to standard questions.

**Initial programme theory**

**Figure 3.**
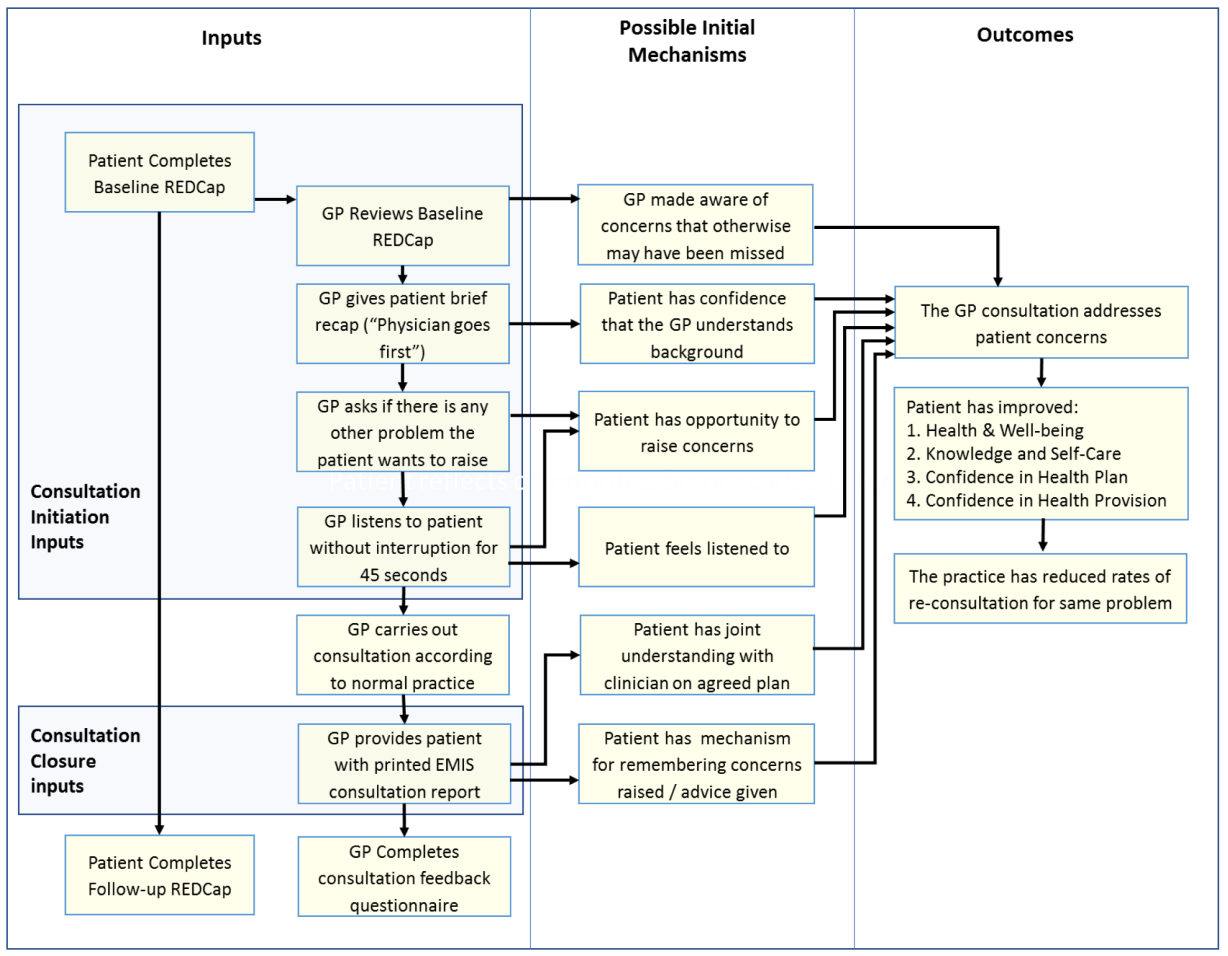
Proposed initial programme theory of COAC.

An initial programme theory of how the COAC intervention was intended to produce outcomes was designed. This was drafted by the study CI and reviewed by the study co-applicants and PPI group before finalisation. This is shown in Figure 3.

***2.4.3 Data collection / measures***

Data collected in the Intervention Development study included clinician and administrator questionnaire data and qualitative interviews. Interviews were carried out by MM and AS and audio-recorded. We aimed for 20 patient interviews, six GP interviews and three administrator interviews.

The purpose of these interviews was to inform development of the intervention through a person-based approach (which takes place in rounds, with the intervention amended at the end of each round). Topic guides therefore focused on the feasibility and perceived usefulness of the pre-consultation form and on the proposed design of the intervention. Patient and GP topic guides are available as open access data (see data availability statement).

Prior to the COVID-19 pandemic, some interviews were conducted face-to-face in the patients’ own homes or other location of their choice, and GP/administrator interviews in the relevant health centre. After March 2020 interviews were conducted by telephone or video link.

***2.4.4 Analysis***

Interviews were transcribed and analysed at the end of each round. The analysis focussed on establishing what changes were required to the pre-consultation form in that round before testing again in the next practice.

To do this, “guiding principles” were established. These are fundamental to the person-based approach, and highlight the objectives of the intervention and the key features that will address context-specific behavioural issues in support of these objectives ^29^. The guiding principles were drafted by the CI, adjusted by the PPI group and agreed by the study co-investigators. A coding framework for changes identified was then established (see Table 1). This framework contained codes to identify the reason for making each change, with reference to the guiding principles and the initial programme theory. After each interview was transcribed, one of the researchers (the qualitative researcher or the CI) listed the possible changes arising from that interview and assigned a code to it. The second researcher then checked this and, if necessary, added new changes to the list, or modified existing ones. The two researchers then discussed any areas of disagreement.

**Table 1.** Coding framework for Table of Changes.

Coding framework		
Code	Stands for	Means
**IMP**	Important for intervention uptake and effectiveness	This is an important change that is likely to impact intervention uptake or effectiveness or is a precursor to that (e.g. acceptability, feasibility, persuasiveness, motivation, engagement), and/or is in line with the programme theory and/or is in line with the Guiding Principles.
**EAS**	Easy and uncontroversial	An easy and feasible change not involving any major design changes. For example, a participant was unsure of a technical term, so a definition is added.
**REP**	Repeatedly	This was said repeatedly, by more than one participant.
**EXP**	Experience	This is supported by experience, for example: 1. PPIs agree this would be an appropriate change. 2. Experts (e.g. clinicians on your development team) agree that this would be an appropriate change. 3. Literature: This is supported by evidence in the literature.
**NCON**	Does not contradict	This does not contradict experience (e.g. evidence), or the programme theory, or the Guiding Principles
**RES**	Research relevant	This is a change to the design of the research, not the intervention
**NC**	Not changed	It was decided not to make this change. Please explain why (e.g. it would not be feasible; or only one person said this).

At the end of each round, the co-applicants all reviewed the table of changes and a final list for the round was agreed. The changes were implemented and the revised pre-consultation form was taken forward to the next round.

This continued for three rounds until a final version of the pre-consultation form was agreed.

### 2.5 Patient and public involvement

This research was informed by PPI both before the study commenced and during the study. PPI contributors received expenses and reimbursement in line with INVOLVE guidance ^32^.

Pre-study PPI was carried out with an existing group that had formed for another study on improving care for patients with long-term conditions ^33^. The pre-study PPI group met three times before the study commenced to advised on the proposal, the process, the pre-consultation form and the consultation summary report. This group was instrumental in the decision to use the PCOQ as the basis for the pre-consultation form and include an option for patients to provide their reasons for consulting.

A new PPI group was convened at the start of the study to include a more diverse membership, involving different ethnic groups and people both with and without long-term conditions. The group then met five times throughout the study. Two of these meetings were specifically to design the pre-consultation form as follows:

**Meeting 1**: In the first meeting, members were introduced to the study and made suggestions on the overall design. They suggested recruiting a range of GP practices with different approaches to appointment booking and extending the study to include Nurse Practitioners.

**Meeting 2**: In the second meeting, members gave detailed input to the pre-consultation form and report before the person-based development started. This resulted in substantial changes to the pre-consultation form which are captured in the Table of Changes (see extended data) and in the summary of changes ( section 3.1.2).

Some members of the PPI group raised concerns that the study could increase health inequity. They were concerned that the recruitment process for the intervention depended on patients firstly having access to a smartphone or computer, and secondly having the ability to complete a questionnaire on smartphone or computer. Despite these concerns, most PPI members felt that proceeding with a digitally-based intervention was acceptable, since if the intervention was useful, work could then be done on improving and extending access.

### 2.6 Feasibility study methods

To provide some context for the realist evaluation, brief details are provided in this paper on the randomization, recruitment and consent and data collection / measures of the feasibility study. This section mainly focusses on describing the data and analysis used for the realist evaluation embedded within the feasibility study ^34^.

***2.6.1 Randomisation recruitment and consent***

In the feasibility study, both the pre-consultation form and summary report were used together in six practices, four randomised to intervention and two to control. Practices who had participated in the Intervention Development study were approached by the CI and new practices were approached by the CRN. Each practice was asked to recruit 30 patients, resulting in 120 in the intervention and 60 in the control (see Table 2).

**Table 2.** Patient recruitment target in control and intervention practices.

	Intervention	Control	Total
**Practices**	4	2	6
**Patients**	120	60	180

General practice administrators searched their practice database using an electronic search strategy which identified patients with upcoming appointments who met the inclusion criteria and sent batch SMSs to patients with a link to the baseline questionnaire hosted on REDCap. The SMSs contained the patient EMIS number and the patient needed to input this so their questionnaire could be identified.

**Figure 4.**
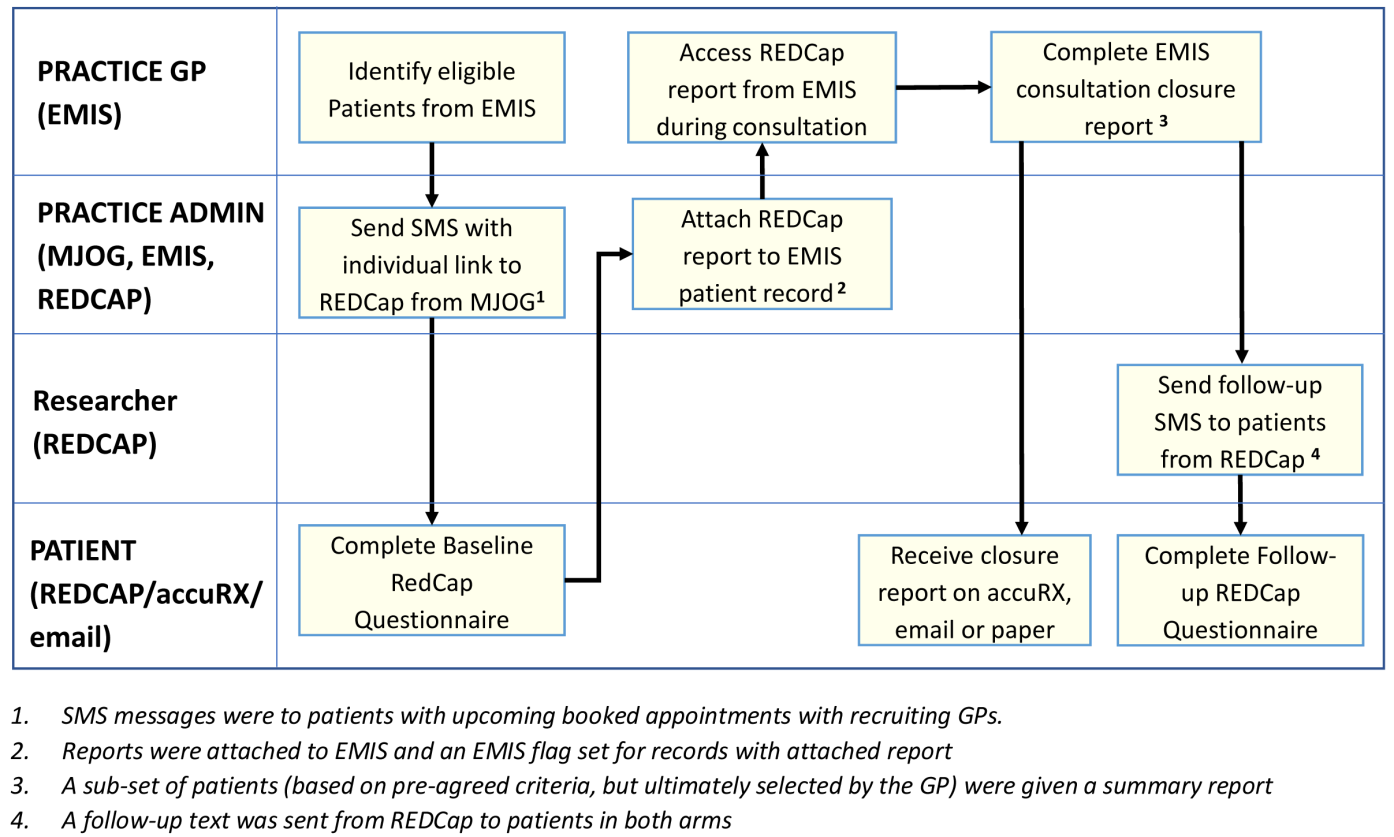
feasibility study workflow: intervention and control arms.

Administrators received an alert when a patient completed a questionnaire. On a regular basis, the administrator downloaded the summary report from REDCap to pdf and attached it to the EMIS patient record system. The baseline questionnaire included an information screen explaining the purpose of the study and how the data would be used. Return of the questionnaire indicated consent to participate in the study. Patients were explicitly asked to consent to their contact phone number being shared with the University of Bristol for the purposes of sending a follow-up questionnaire. Further consent for use of that phone number to contact the patient for interview and for access to the patient’s record for demographics and re-consultation rates was requested in the follow-up questionnaire ^35^. A similar approach has been taken for a number of other cluster trials ^35– 37^. The researcher then took informed for recording the interviews and use of anonymised quotations in publications prior to the interview itself. This consent was written for face-to-face interviews and audio-recorded for telephone interviews. Before the start of the COVID-19 pandemic, all consent was written. The ethics committee approved an amendment to collect audio-recorded consent for patients interviewed during the pandemic. A workflow for this is shown in Figure 4.

***2.6.2 Data collection / measures***

Feasibility study data included clinician and administrator questionnaire data, interview data, and quantitative patient data. The quantitative patient data is described in the feasibility study linked paper ^34^. Interview and questionnaire data was collected as follows:

**Clinician questionnaire data:** The GP questionnaire requested information for each consultation (new/review), modality (face-to-face, telephone or video), whether the pre-consultation form was useful, and why a summary report was used.

**Interview data:** Interviews were conducted by the CI and the project research associate. Topic guides were designed to inform a realist evaluation and therefore focused on the outcomes that patients/GPs perceived, the mechanisms by which these were achieved and the contexts. Patients and practitioners were interviewed to the point of achieving “theoretical sufficiency”, i.e. when the data analysis has yielded one or more coherent theories which are relevant to the study aims ^38^. Interviews were conducted by phone and audio-recorded.

***2.6.3 Analysis***

Realist evaluation seeks to explain the complex relationship between context, mechanisms and outcome. The explanatory proposition of realist evaluation is that interventions work (i.e. have successful outcomes) only in so far as the individuals involved take up ideas and opportunities (mechanisms) within the social and practical conditions in which they are operating (contexts) ^39^. This is then reported in terms of contextual factors (What elements of the intervention work, for whom, in what consultations?) and content-mechanism-outcome configurations (CMOCs). A CMOC is a hypothesis that the program works to produce an outcome (O) because of the action of some underlying mechanism (M), which only comes into operation in particular contexts. (C)

The realist evaluation used the interview data collected in the feasibility study supplemented with the interviews from the intervention development study. To carry out the realist evaluation, the CI (MM) read and re-read the initial interview transcripts from both patients and practitioners, in order to gain an overall view of the accounts given and to identify patterns in the data. She then revised the programme theory and devised an initial set of CMOCs. The research associate (AS) independently developed three lists of context, mechanisms and outcomes. These were cross-checked against the CI’s CMOCs which were then revised and detailed evidence presented against each of them. An experienced realist evaluator (GW), then read through the detailed evidence and the final CMOCs were agreed in collaboration. Four researchers (MM/AS/GW/CS) reviewed the realist evaluation and programme theory before finalising.

### 2.7 Sponsorship, funding and ethical arrangements

This study was sponsored by the University of Bristol. Ethics approval was granted by Frenchay Research Ethics committee ^40^ and the Heath Research Authority (HRA). BNSSG Clinical Commissioning Group Research and Evidence Team provided research and development approval. The study was NIHR funded and supported by the NIHR Clinical Research Network who liaised with centres on the researchers’ behalf.

Insurance was provided by the University of Bristol as research sponsor. The study sponsor and funders did not have any role in study design; data collection, management, analysis, and interpretation of data; writing of the report; or the decision to submit the report for publication.

The Feasibility study was registered in the ISCTRN registry (ISRCTN13471877) and on the CRN portfolio (42005). The study protocol was published before recruitment completed ^41^.

## 3 Results

### 3.1 Intervention development

***3.1.1 Participants***

Table 3 shows the number of recruits to the Intervention Development study. Three practices were recruited from the top, middle and bottom of the index of deprivation (IMD) score, where 1 indicated a high level of deprivation and 10 a high level of affluence. Each practice had two participating GPs. One of these was an advanced nurse practitioner, but for simplicity has been referred to throughout as a GP. Each practice recruited their target of 15 patients, which was 6 to 9 per GP. We had intended to interview 20 patients from the 45 recruited but were only able to interview 12.

**Table 3.** Development of the pre-consultation form: GP and patient recruits.

Recruiting practice	Practice IMD score	Date	# Recruiting GPs	# Admin staff involved	Patients recruits per practice (target = 15)	Patient interviews per practice (target = 6 to 7)	Response rates (SMS sent / Patient recruits)
Practice 1	9	Dec 19	2	2	15	6	15%
Practice 2	5	Feb 20	2	1	15	2	17%
Practice 3	1	Nov 20	2	2	15	4	50%
Total			6	5	45	12	

**Table 4.** Guiding principles – pre-consultation form.

Intervention Design Objectives	Key Features
To make the pre-consultation form appealing for patients to complete	▪ Convincing text message and initial screen ▪ Targeted at the right kind of patient ▪ Using positive language throughout
To create a positive and beneficial experience for patients completing the pre-consultation form	▪ Easy to complete ▪ Seems relevant to patient ▪ Ensuring the intervention provides something interesting, relevant, and helpful for the user (patients)
To make the report as useful as possible for GPs	▪ Relevant information on the form ▪ Clear and easy to read form ▪ Access and reading of the form fits within normal process of the consultation ▪ Patients provide optimal amount of info and the right kind of info
To create a positive and beneficial experience in the consultation, where the pre-consultation form promotes communication and problem solving	▪ GP training and ongoing support ▪ Ensuring the intervention provides something interesting, relevant, and helpful for the user (patients and GPs) ▪ Reciprocating intervention usage by providing immediately rewarding feedback

***3.1.2 Summary of changes***

The person-based approach relies on a set of guiding principles. These were agreed in advance and informed the intervention development by highlighting the objectives of the intervention and the key features that will address context-specific behavioural issues in support of these objectives ^29^ ( Table 4).

Table 5 summarises the key elements of the pre-consultation form that were changed over the 3 rounds. As shown in Table 5, the form was shortened during the process and the wording clarified. Minor wording changes were added to improve uptake and encourage patients to add more free text. Diffusion of innovation theory was used to inform this. The report instructions and training for GPs were adjusted to emphasise how important it was for GPs to let the patient know they had read and understood the report. Administrative processes were simplified.

**Table 5.** Summary of changes: pre-consultation form.

	Issue identified	Feature added
**PPI / patients**	The validated questionnaire used (PCOQ ^42^) was too long and repetitive. It felt like a research tool, not for practical use.	The questionnaire was simplified and reduced in length. Some of the question wording was adjusted to gear it more towards practical use.
**PPI / patients**	Further information on each question is asked for at the end of the form. It would flow better if this was asked for throughout.	The form was adjusted so that additional questions asking for more information appeared immediately a patient gave a low score.
**Patients**	It was very important to patients that GPs mentioned they had read the report.	Emphasised this in training, and added “remember to tell the patient you have read this report”
**Empirical**	In the first practices, just over 15% of patients who were sent a message responded.	After the first two practices, added into the SMS: “Completing this form should really improve your experience of your appointment.” (diffusion of innovation theory technique)
**GPs**	The report is more useful when the patient adds free text, especially for health concerns and support.	Final version flagged to patients those questions where it was really important the patient put in additional information.
**GPs**	To simplify the report for GPs, patient responses were summarised and some were merged. Some meaning was lost in this: e.g. “adherence to medication” and “adherence to healthy lifestyle” are two distinct ideas which it was confusing to merge on one line.	The final report contained 10 lines. Most pertained to a single question, and the captions for these, although summarised, attempted to follow the language of the question patients responded to. Three pairs of questions were still merged, but GPs confirmed these were clear.
**GPs**	It was not fully clear when the free text is system generated and when it is written by the patient	Added a line to GP report **“ *Blue signifies free-text written*** ***by the patient.*** Black text is based on patient’s selected responses.”
**GPs**	Patients sometimes give multiple reasons for attending all on one line, which is difficult to read. Some are existing problems and some new, so it would be useful for patient to indicate if and how many times they have consulted about a problem before.	Ask patients for their reasons for attending at the start of the form, stating that they will be able to enter up to three reasons (so that they are split out into separate lines for each reading). Also allow patients to indicate whether they have consulted about the problem before
**GPs**	GP raised in training sessions that some of the reports are very detailed and might be too time-consuming to read.	In practice, reading a report took about 20 secs. A timed reading to demonstrate this was incorporated into the GP training.
**GPs**	Initially, administrators sent a task to GPs and added a pop-up. GPs felt this was unnecessary and would prefer a note in the appointments book.	Agreed that the words “COAC patient” should be added as a note in the appointments book after each COAC Study patient appointment
**Admin**	The administrative process of generating an individual link for each patient was time-consuming	The process was adjusted so that patients input the EMIS number themselves. Administrator then sent the same link to each patient.
**Admin**	Some patients completed the form immediately before their appointment, and admin did not have time to add it to the record.	Added into the SMS: Please complete the form today so that your GP/nurse has time to read it. Sent a notification to the administrator each time a report is completed on REDCap

Table 5 is a summary of 32 changes documented, agreed and made during the three rounds. The full table of 32 changes, including verbatim quotes and coded rationale for making each change are available as open access data (see data availability statement).

**Figure 5.**
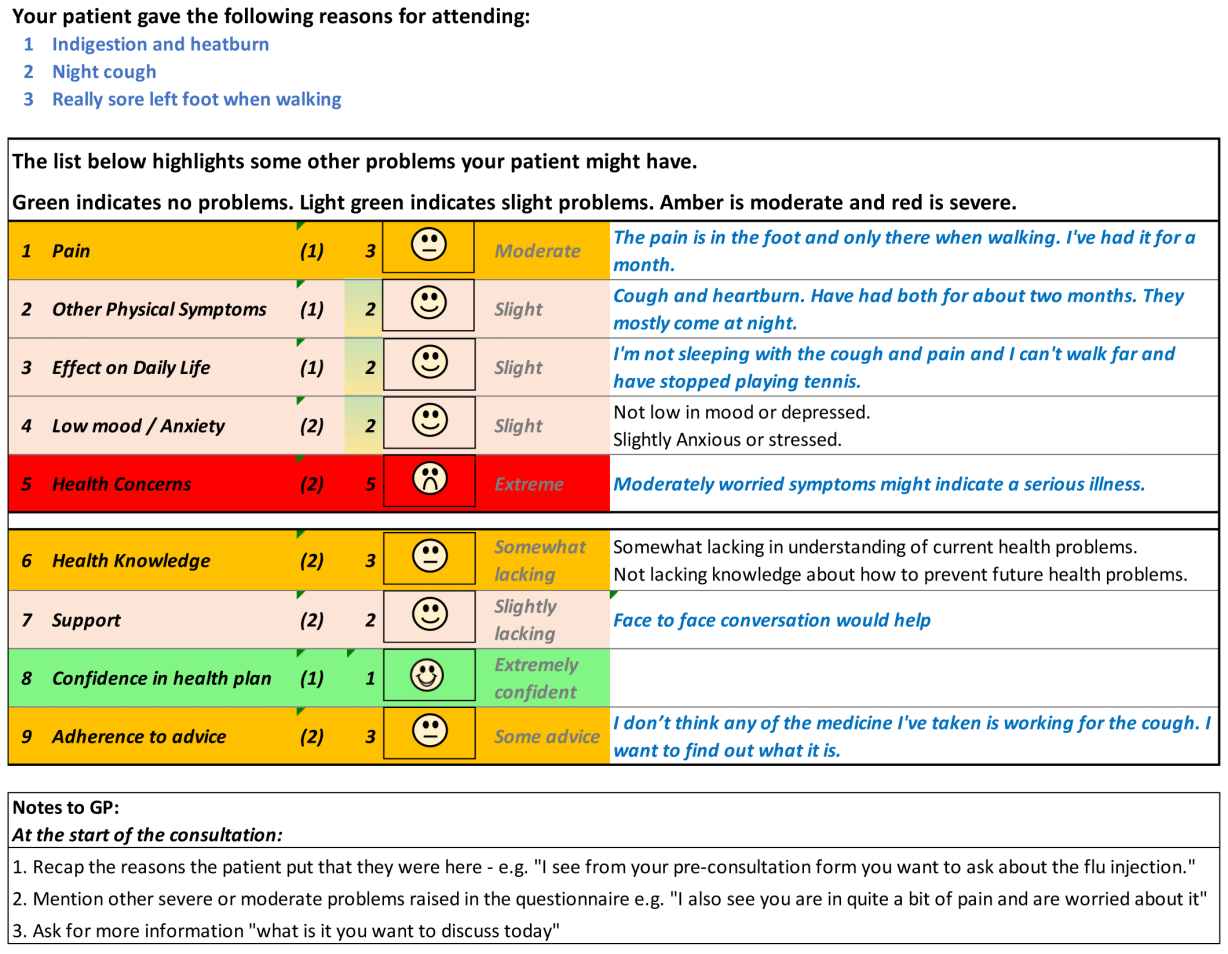
Pre-consultation report: Pilot version (start of intervention development).

**Figure 6.**
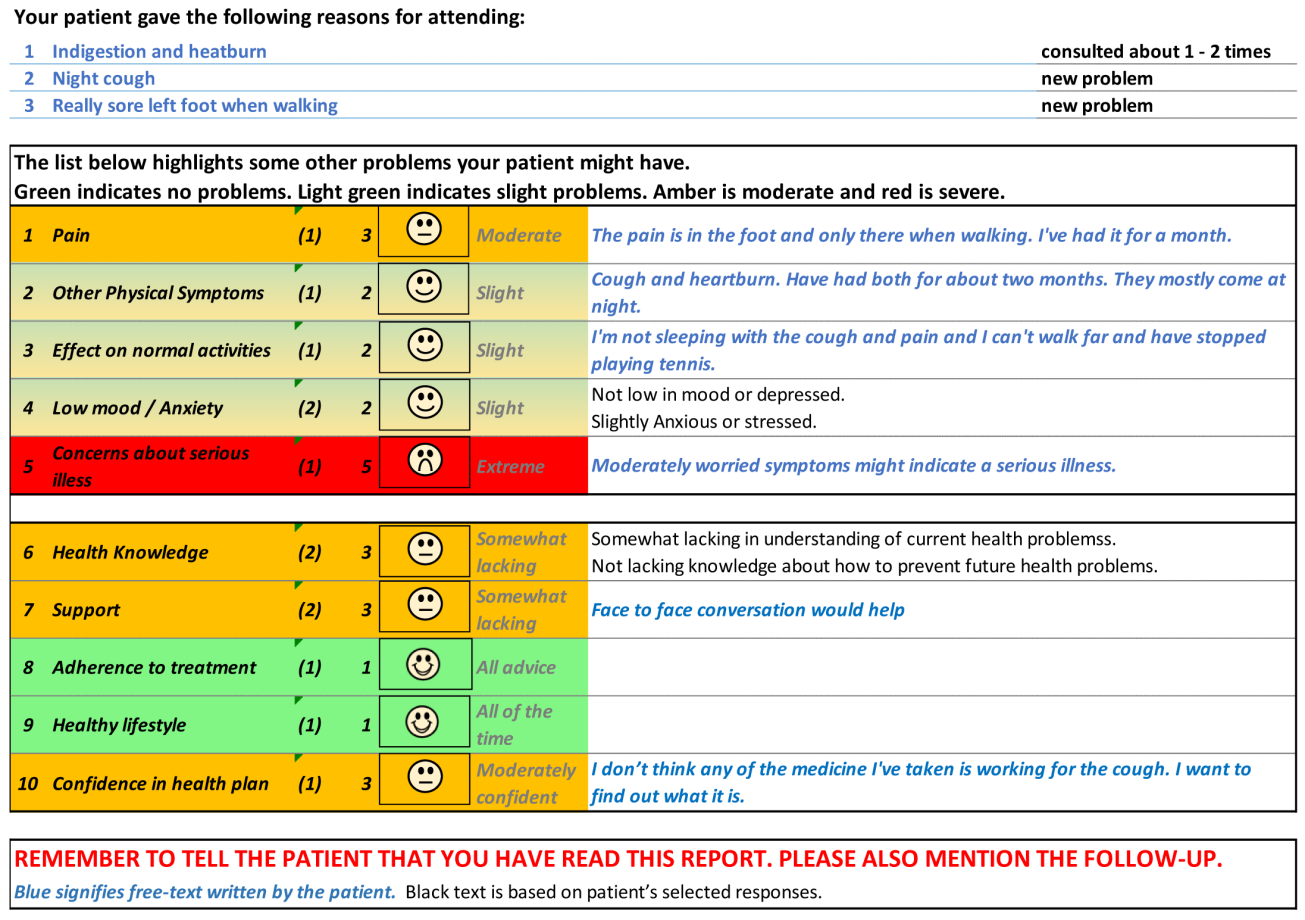
Pre-consultation report: Final version (end of intervention development).

Figure 5 and Figure 6 show the initial (start of round 1) and final (end of round 3) pre-consultation report seen by GPs.

**Figure 7.**
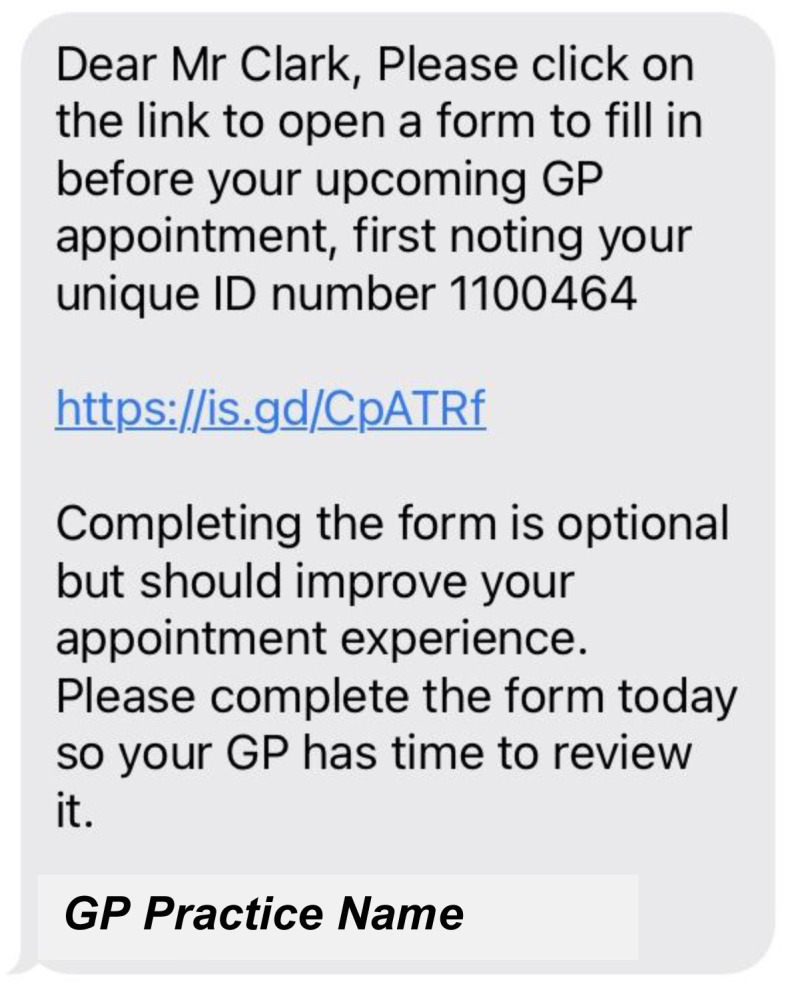
Pre-consultation form: Patient SMS message received.

**Figure 8.**
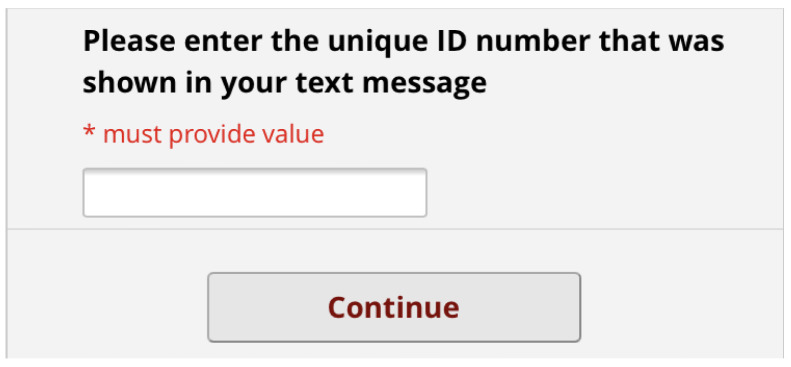
Pre-consultation form: Opening Screen.

**Figure 9.**
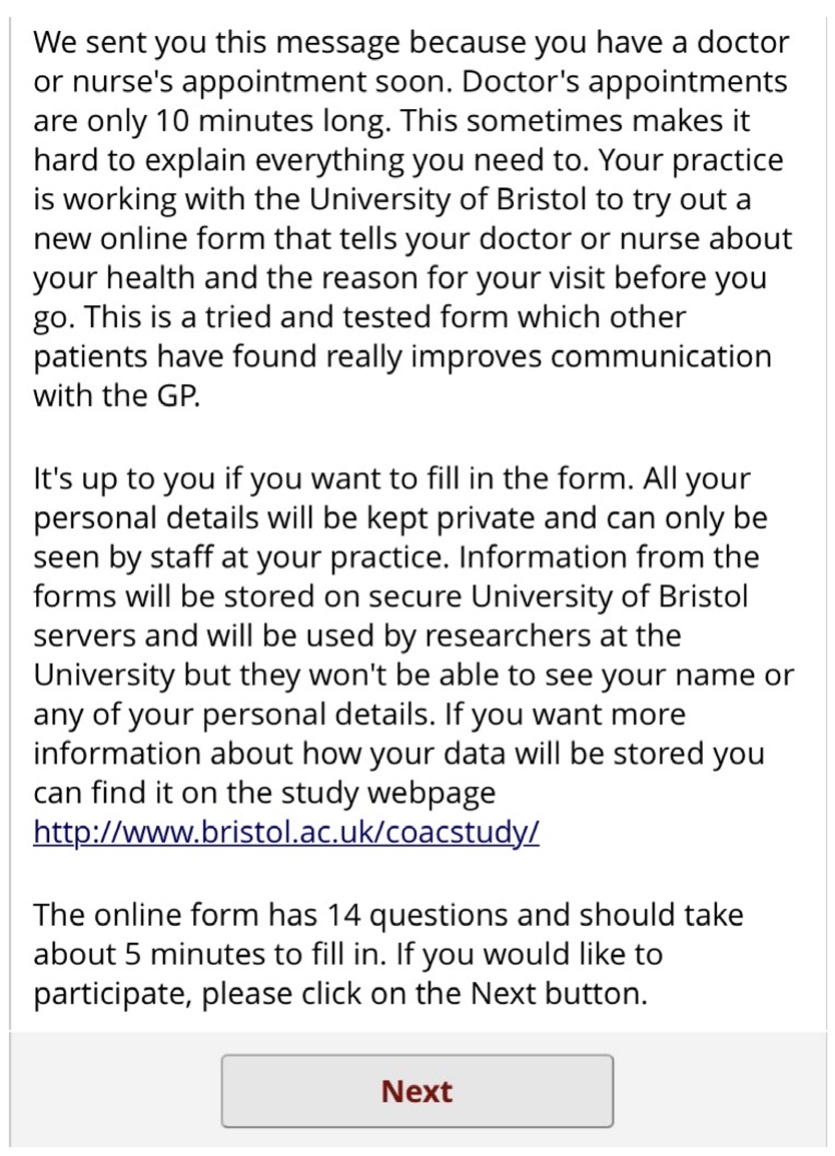
Pre-consultation form: Information and consent screen.

**Figure 10.**
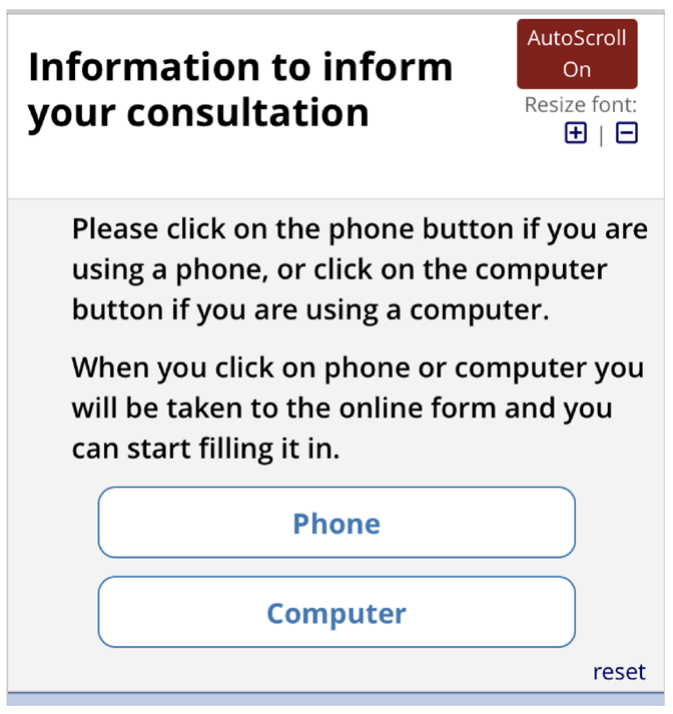
Pre-consultation form: Selection of phone or computer.

**Figure 11.**
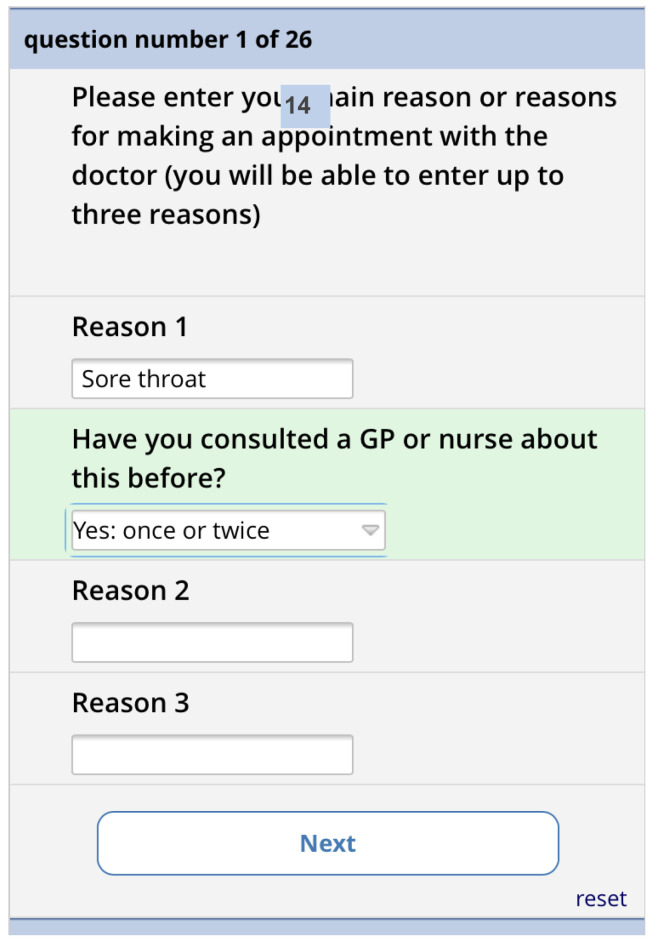
Pre-consultation form: Reasons for consultation screen.

**Figure 12.**
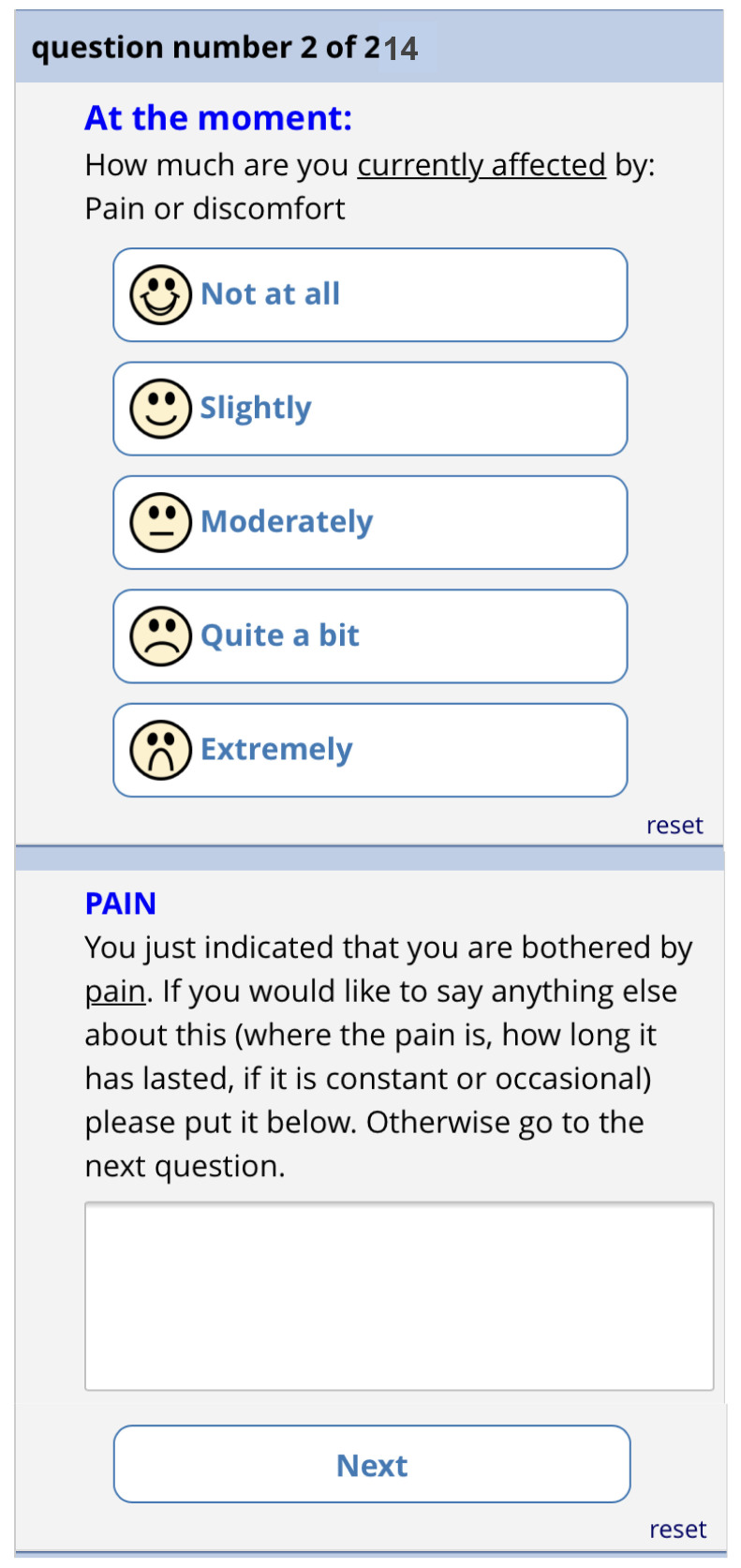
Pre-consultation form: Pain question.

Figure 7 to Figure 12 show the final pre-consultation form from the patient point of view. The patient receives an SMS on their phone with a link to the form and their unique ID number to enter in the first screen ( Figure 7). This form lets the patient know that completion is optional but should improve their appointment experience. On clicking the link, the patient has to enter this ID number before proceeding ( Figure 8). The patient then sees a screen of information about the study. Again, this screen encourages the patient to complete the form by saying other patients have found it useful. It also contains a link to the study website for more detailed information ( Figure 9). If the patient clicks *Next*, they are asked if they are completing the form by phone or computer ( Figure 10). Depending on what they select, they are shown a matrix version of the form (computer) or one item per screen (phone). The first screen asks for the reasons for consulting and how long the problems have persisted ( Figure 11). There is then one screen for each of the 13 remaining questions. Each question has the same 5-point categorical response scale. Figure 12 shows the phone version of the first question for “pain”. If the patient responds with anything other than “not at all”, a free text box appears asking them for more information. This question populates the first row in the report that goes to the GP (shown in Figure 6). The remaining 12 questions follow a similar format and are also used to populate the report, with the extremity of the response given by the patient driving the colour of the row.

**Figure 13.**
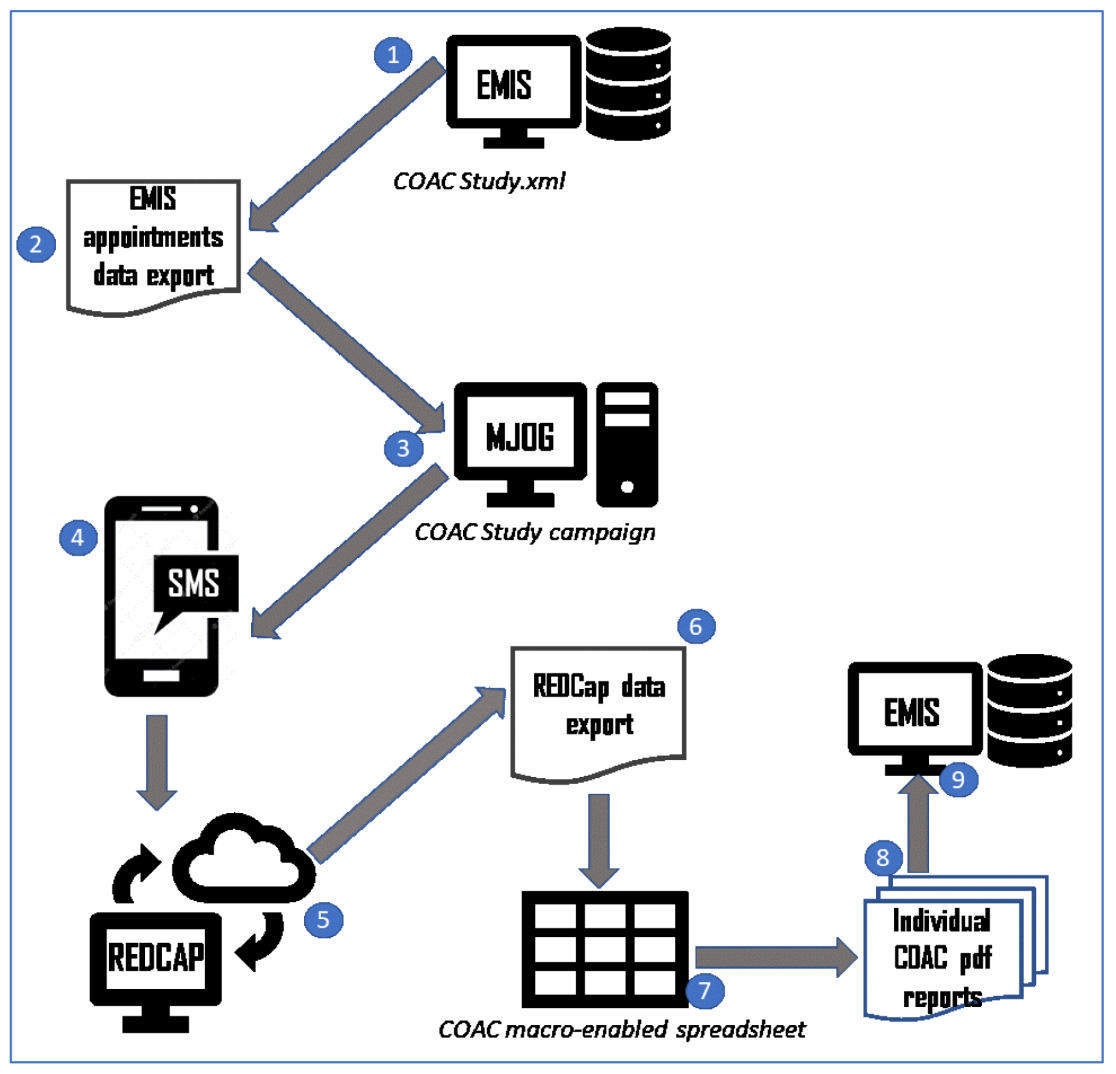
pre-consultation report generation: process diagram.

The final agreed administrative process is shown in Figure 13.

**1.    The administrator** runs a pre-built report in EMIS which identifies patients with upcoming appointments with recruiting GPs.

**2.    The administrator** exports the list of patients to a csv file.

**3.    The administrator** opens MJOG, imports the list of patients and sends an SMS with the link to each patient.

       ----------------

**4.    The patients** receive an SMS with a link to the pre-consultation form on their phone.

**5.    Recruited patients** give consent, complete the form and the data is stored in REDCap.

       ----------------

**6.    The administrator exports data from REDCap** to a csv file. 

**7.    The administrator** runs a macro to generate an individual pdf file for each patient.

**8.     The administrator** attaches each pdf file to the patient record and adds a note to the appointment book for that patient to alert the GP that the report is there.

       ----------------

**9.     The GP** reviews each patient report on EMIS before the consultation and carries out the COAC intervention with that patient (summarising the information for the patient, asking if there is anything else, listening without interruption, providing a report on closure for a subset of patients).

### 3.2 Realist evaluation

***3.2.1 Participants***

Forty-five interviews were carried out in the feasibility study: 30 patients, nine GPs and six administrators. In addition, the eighteen GP and patient interviews from the intervention development phase were also used to inform the realist analysis. Interviews at each site in each phase are shown in Table 6.

**Table 6.** Patient and practice interviewees for the pre-consultation form.

	Intervention Development	Feasibility Study
*Patients*		
Site 1	*6*	*6*
Site 2	*2*	*7*
Site 3		*9*
Site 4		*8*
Site 5		
Site 6	*4*	
*GPs*		
Site 1	*2*	*2*
Site 2	*2*	*2*
Site 3		*2*
Site 4		*1*
Site 5		*1*
Site 6	*2*	*1*
*Administrators*		
Site 1		*1*
Site 2		*1*
Site 3		*1*
Site 4		*1*
Site 5		*1*
Site 6		*1*
**Total**	***18***	***45***

*Sites 1, 2 and 6 were in the Intervention Development Study as well as Feasibility. Sites 1 to 4 were intervention sites and sites 5 and 6 were control sites.

In the qualitative analysis which follows Patients 1 to 20 are from the intervention development study and patients 30 to 50 from the Feasibility study. So that the evolution of their views can be compared, the same identifier is used across the studies for GPs who were in both studies.

***3.2.2 Summary findings from the process evaluation***

The process evaluation of the feasibility study is presented in the linked paper ^34^. A key finding of this evaluation was that the pre-consultation form and summary report are useful for different types of patients and consultation and each intervention results in different outcomes, triggered via separate mechanisms. It was therefore more appropriate to carry out a separate realist evaluation for the pre-consultation form and the summary report respectively than to update the initial joint programme theory which was shown in Figure 3.

The feasibility study also found that the pre-consultation questionnaire was useful to patients and GPs and took very little of their time. However, the technical process surrounding the pre-consultation form required too much support from the research team to be easily rolled out. The realist evaluation should be read in this context.

***3.2.3 Revised programme theory***

**Figure 14.**
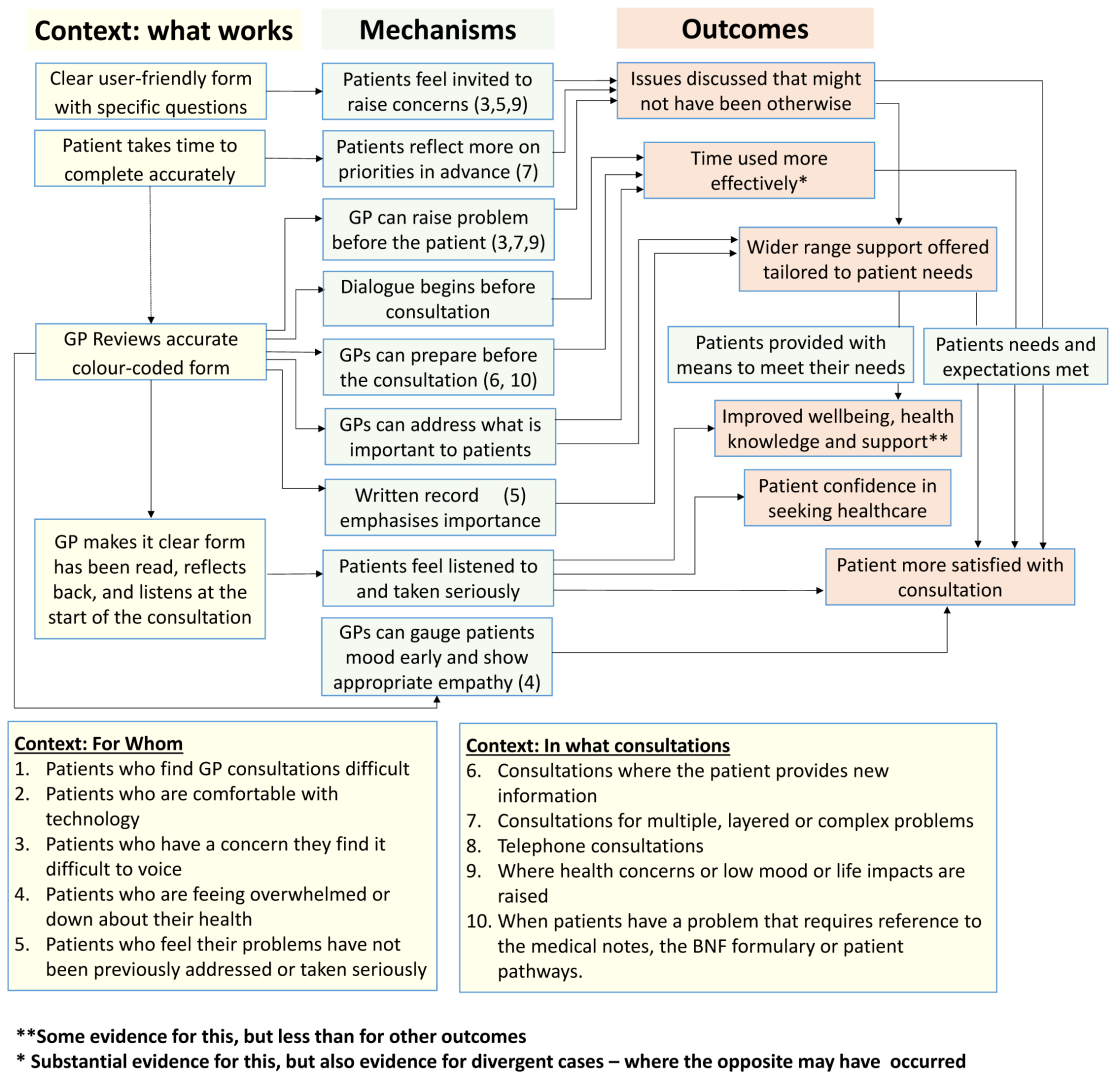
Pre-consultation form, revised programme theory.

Our analysis of the data from 63 interviews enabled us to revise the programme theory for the pre-consultation form. This is shown in Figure 14. This presents fourteen context-mechanism-outcome configurations (CMOCs) identified in the data in a single diagram showing interlinked context (what works), mechanisms and outcomes. “What works” can be understood as how the interventional subcomponent needs to be implemented (to alter context) to achieve the outcomes. The mechanisms are processes that are ‘triggered’ by the context to cause outcomes. Some mechanisms are only activated for certain types of patients and consultations. This information on which types of patients and consultations are shown by the numbers in brackets in the green mechanism boxes. Two of the outcomes are more distal than the others and for these, the context in which they are achieved is represented by another outcome in the programme theory (functioning as a context for that CMOC).)

***3.2.4 Context (what works, for whom and what circumstances)***

This section focuses on providing an overview of the context through which the mechanisms are activated using a realist evaluation framework of “what works, for whom, in what circumstances”. Details about the CMOCs in which these contexts function may be found in below in section 3.2.5.

**3.2.4.1 What works**

Key elements of the pre-consultation form that worked well were: 1) the simple format of the form combined with the ability to add more information where required 2) patients taking time completing the form accurately and with the right amount of detail 3) the colour-coded format of the report 4) GPs ensuring that they read the report properly 5) GPs letting the patients know they have read the form and then listening.

The form was most useful for GPs when patients took time to complete the form accurately and provided an appropriate amount of detail, providing free text for an item if they scored the middle option or lower. This was particularly the case when it was a health concern or a need for information or support. 

GPs found the colour-coded fixed format traffic light system worked well as it made it easy for them to quickly read and assimilate the information.

*It’s certainly much quicker for a clinician to look at than an [practice electronic triage system] is. You just really want your eyes to go straight to, what it is that matters, and the traffic light system enables you to do that and getting engaged. **(GP 1, Feasibility Study)***

The colour-coded format was occasionally mis-leading, when the patient had a chronic condition which involved chronic pain, physical symptoms and/or anxiety, but that was not necessarily what they wanted to talk about that day. GPs normally established this quickly at the start of the consultation. Many patients tend to select the “slightly” option for mild ongoing problems that they don’t need to discuss with their GP and if this was selected but the patient had not put any more detail, it worked best if the GP did not raise that in the consultation as an issue.

GPs letting patients know that they have read the form at the start of the consultation was very important to patients, and a driver for patient perceiving that the GP was prepared and feeling listened to and taken seriously. One patient contrasted this approach with the previous approach of the GP greeting the patient and asking how they can help.

*I felt like I wasn’t going in blind because normally, you know, I remember before COVID you’d walk in and GP would look at you like, okay, like why are you here? Even though you’ve spilled your life out* to the *receptionist? [laughs] They still haven’t got a clue while you’re there…. So, you think, why were you asked all these bloody questions when either it’s not been relayed or they’ve not read the notes. (Patient 27, Feasibility Study)*

One GP noted that she had also experienced this reaction from patients.

*Well I think it’s just that acknowledgement, because when you say… normally in the consultation I say, ‘Hello, it’s Dr Jones returning your call, how can I help today? And sometimes they’ll be a bit grumpy, they’ll say, ‘Well, I told your receptionist.’[…] or they’ll say, ‘Well, you’ve got my notes in front of you haven’t you?’ [Laughs] so I think this just was a way of acknowledging to patients that you were interested in them, and that you had read what they had written. (GP 5, intervention development study round 3)*

Although the majority of patients said the GP made it clear they had read the form, one or two did not think it had been read. Of the patients who though the form made no difference to their consultation two of these were unsure that the GP had read the form. This underlines the importance of the GP making it clear that they have read the form, by reflecting back the patient’s words to them, or bringing up issues before the patient does.

**3.2.4.2 For what type of patient**

The pre-consultation form works best for: 1) Patients who find GP consultations difficult 2) patients who are comfortable with technology 3) patients who have a concern they find difficult to voice 4) patients who feel overwhelmed or down about their health 5) Patients who feel their problems have not been previously addressed or taken seriously. The last three of these are specific to particular mechanisms and are covered in 4.3.3 (CMOCs). The first two (patients who find GP consultations difficult and who are comfortable with technology) apply to the intervention generally. One patient described how she felt about GP consultations:

*Because when you go to the doctors it’s really quite nerve-racking, a nerve-racking experience. So actually being able to write something down and have a little bit of time to think about it, like your symptoms and what you’re feeling and everything else was really helpful to me. (Patient 28, Feasibility Study)*

Many of the patients who were interviewed commented that they were comfortable with technology:

*I’m quite used to sending things, bank details and all kinds of stuff by my phone […] I’m aware some people might not, but I do feel fairly secure, rightly or wrongly, with that kind of thing these days. So it didn’t really, I have to say, bother me too much. I wasn’t worried about it. (Patient 23, Feasibility Study)*

We were unable to interview non-responders, so do not have information on what types of patient did not complete the form, but practice administrators were able to see the age profile of the patients who they messaged and had visibility of who responded and some of these felt younger people responded.

**3.2.4.3 In what types of consultations**

The pre-consultation form was most useful for consultations 1) where new information was raised, 2) where complex, multiple or layered problems and situations were discussed, 3) which were conducted by telephone 4) where information about health concerns, low mood or impacts on life were raised, or 5) where the problem required reference to the BNF, funding rules or the patient history. The last two of these are specific to particular mechanisms and are covered in 4.3.3 (CMOCs). The first three (new information, complex problems and telephone consultations) apply to most of the CMOCs and are discussed below.

GPs felt the form was most useful for new problems. The practice appointment booking procedure followed by Site 1 meant that a disproportionately high number of patients who had follow-up appointments booked were sent the form. This practice had also been involved in the Intervention Development study before the practice booking policy changed. The GP commented:

*Last time [Intervention Development], we had a lot of new patients, and it worked really well, because they were not such follow up things. Logistically, it was fine [this time]. But just from a quality of information, it probably wasn’t as useful, I don’t think. (GP 2, Feasibility Study)*

Some patients agreed with this:

*For me, particularly, I don’t think it made any difference to the way the GP listened. It was pretty much the same as my normal consultations, but I do think if it was a new problem, then it would be more helpful. (Patient 11, Intervention Development Study round 3)*

This patient was consulting about a problem they had seen the GP about several times before, and didn’t have any new information to raise, so found the form less useful. 

Patients who had complex problems, multiple problems or problems with different aspects to them found the form very useful. Few people with single simple problems completed the form, but when they did, they found the form less useful:

*They [the questions on the pre-consultation form] weren’t relevant to me because I mean to be fair all I was asking was a very simple question. Because it’s one of those things where literally a two-minute conversation was all I needed. (Patient 10, Intervention Development Study round 3)*

**Figure 15.**
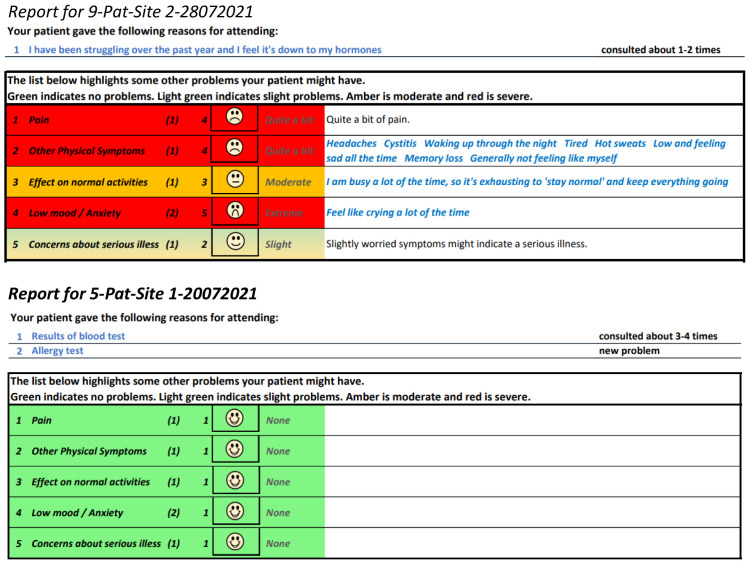
Excerpt of pre-consultation report for different types of problems.

Some GPs, on the other hand, still found the forms for these very simple problems useful, because they highlighted that there was no hidden agenda to be concerned about and sped up the consultation (see CMOCs). Figure 15 shows an example of a report where the patient found the consultation useful, next to one where the patient did not find it useful. As the figure shows, the most useful consultation was one where multiple aspects of the problem were raised. The least useful was for a very simple problem.

Some patients found the pre-consultation form more useful over the phone because communication is harder than in a face-to-face consultation so the form acts as an aid to communication.

*Definitely, definitely, [more useful over phone] because obviously the phone, you’ve got the delays and we were both using … well I was on a mobile phone when she contacted me - there’s always that sort of delay in speech. And I felt that she was able to read what I’d put prior to actually speaking to me, so she understood better. So I felt from the telephone interview point of view the form worked very well. (Patient 2, intervention development study round 1)*

This patient felt that the GP understood better because she had read the report, and therefore the delays in the phone were less disruptive that they would have been otherwise. Some GPs and patients, however, thought it made no difference whether the form was used over the phone or face-to-face.

***3.2.5 Context mechanism outcome configurations (CMOCs)***

In this section, more details are provided on the fourteen CMOCs shown in the programme theory ( Figure 13).

**Outcome: Issues raised that might not have been raised otherwise**

*CMOC 1: Invitation to raise concern->Issues raised that might not have been*

When patients who have a concern they find difficult to voice receive a clear user-friendly form with specific questions about their problems (C), issues are sometimes discussed that might not have been otherwise (O) because the patient feels invited to raise their concern (M). ( Box 1)

**Box 1. CMOC 1 diagram**

Patient has a concern they find it difficult to voice (e.g. a health concern or low mood or are worried they won’t be taken seriously) are given a …

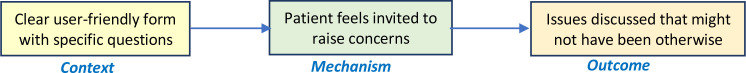

Because they were explicitly asked on the form, patients who had concerns they found it difficult to voice found it easier to raise issues, including low mood, health concerns, life effects and support needs. One patient, who was expert in her own condition (lupus) had been worried the pain in her side might be liver related, but hesitant about raising this for fear she “looks like you’re trying to tell them [the GP] what they should know”:

*So with lupus […] you’re always second guessing what might be going on underneath. […] Sometimes you don’t want to say that necessarily outright […] with doctors, you think, oh, if it looks like you’re trying to tell them what they should know. I know enough to know that lupus affects the liver. I know enough to know my blood test shows that I’ve got abnormality in my liver. So in the back of my head, there’s almost a little bit is this discomfort I’m having for the last six months that I’ve not mentioned to anybody, is this a reason why I’ve now got this abnormal blood test in my liver? […Now] because I’ve written it on the form, she could actually say, ‘Oh, I know you mentioned it’s possibly the liver. Actually, it’d be extremely rare for anybody to actually have pain in the liver like this. It would be very, very rare. So I think we need to keep an eye on it. If it keeps getting worse, then we’ll need to have a scan’ [… ] So it made a huge, huge difference, so yeah. (Patient 23, Feasibility Study)*

This patient had a health concern which for six months she had avoided explicitly asking the GP about. The invitation to share health concerns on the form meant this was raised and addressed in the consultation. GPs also noted that there were things raised on the form which may not have been had the patient not been invited to share them:

*So things around sort of suicidal planning […]; I don’t know whether they would have told me those things in the consultation or not. So it maybe that those things wouldn’t have come out if they hadn’t written them down […] it definitely did sort of ring alarm bells that hadn’t been there when I spoke to the patient previously I suppose. (GP 7, Feasibility Study)*

This GP was referred to a patient who had disclosed significant suicidal planning. The GP said this level of planning had not been evident when he last spoke to the patient, and he was not sure if the patient would have disclosed all the details without being asked to give more information on the form.

*CMOC 2: Greater patient reflection->Issues discussed that might not have been otherwise*

If a patient has multiple, layered or complex problems and this patient takes time to complete the form accurately (C), issues can be discussed that might not have been otherwise (O) because the patient reflects more on what their concerns and priorities are in advance (M). ( Box 2)

**Box 2. CMOC 2 diagram**

Consultation about multiple, layered or complex problem and …

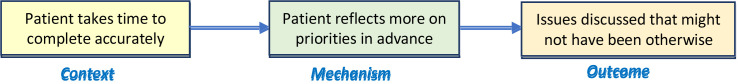

Some patients felt like the action of completing the form made them reflect on why they wanted to see the GP and prepare better for the consultation. Patients explained this as follows:

*I had multiple things that I wanted to talk to her about, multiple symptoms, and often you can forget whereas it made me actually think, ‘well, actually I want to talk about this. ‘I want to talk about that’, so it made me better prepared. (Patient 12, Intervention development study round 3)*
*It makes you think carefully about why you know why you are bothering the doctor if you like or yeah it focusses your mind on your problems. (Patient 33, Feasibility Study)*

The first patient above had multiple symptoms and the action of the form helped her reflect on what she wanted to discuss and prepare what to say. The other patients had a problem with different layers to it; pain that was affecting her sleep, quality time with her family, was causing low mood and she was also worried about the underlying cause.

Some GPs also felt that patients were more prepared in the consultation. One GP described her patients as more “focussed”. This GP acknowledged that this was “my perception of their perception”; i.e. the GP could not say categorically whether her patients were more prepared, only how prepared the patient seemed to her.

There were also a substantial number of patients who thought completing the form did not necessarily make them better prepared, because they tended to prepare for a GP consultation naturally anyway, and the form appealed to them because it was a mechanism for doing that in a way that could be shared with the GP.

*No, I didn’t [feel the form made me more prepared] if I’m honest […] because on those occasions when I do see my GP, I always make a note anyway as I would if I was writing a letter to the bank or anything. I’m a writing down sort of person, so I’d make a few notes anyway, so that’s what I do and in all honesty, that’s more about the kind of person I am. (Patient 40, Feasibility Study)*

*CMOC 3: GP can raise problem before patient->Issues discussed that might not have been otherwise*

If a patient has a concern they find it difficult to voice, low mood or a health concern, or multiple, layered or complex problems and this patient completes the form accurately, and this accurate colour-coded report is shared with the GP (C), issues can be discussed that might not have been discussed otherwise (O) because GPs have a written record to refer to in the consultation so they can raise the concerns themselves if the patient forgets (M). ( Box 3)

**Box 3. CMOC 3 diagram**

Patient has concern difficult to voice, low mood, health concern or complex / layered problem and …

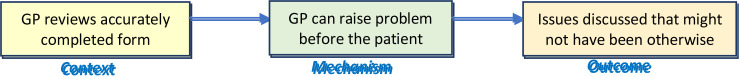

Some patients liked having the written record because GPs could bring up problems before the patient had to. One patient explained that she was worried her symptoms might be cancer and the form helped her express this:

*I think everyone’s been in the situation where they go to a doctor to talk about something that they find hard to talk about or they might find it difficult to voice their concerns. […] in the form I filled in this time it said what are you really worried about, and I was able to write down the word cancer because that’s something that worries me, worried that I won’t be here for my children, if I had to say that to the doctor I’m sure I would’ve just burst into tears saying it and then I wouldn’t have been able to have a particularly productive consultation. But because I was able to write it down before and she knew what was on my mind and I knew she knew what was on my mind, she took the consultation really seriously and was very clear about the steps she was going to take. (Patient 38, Feasibility Study)*

This patient found it difficult to voice her concern but the form invited her to share what her concern was. This then led to this issue being addressed in the consultation. The GP took her concerns seriously and put together a plan of action, which included a fast-track cancer referral.

GPs also commented that it was useful to be able to raise concerns before patients did, in particular for sensitive problems. One GP gave the example of sexual health:

*there was one around sexual function, potentially, that is quite a difficult thing for someone to start talking about in a consultation. Probably would have got there in the end, but it just allowed that to come out much more easily, and without as much difficulty or embarrassment on anyone’s part, I think. So, I think it is really useful for those kinds of things, that it’s something that’s slightly sensitive to bring up. (GP 2, Feasibility Study)*

**Outcome: Time used more effectively**

*CMOC4. Dialogue begins before consultation->time used more effectively*

When patients complete the form accurately and this accurate colour-coded report is shared with the GP (C), time can be used more effectively (O) because the dialogue begins before the consultation (O) ( Box 4)

**Box 4. CMOC 4 diagram**

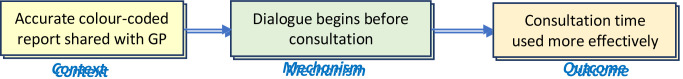

Some patients commented that the GP reading the form made the consultation more efficient because the dialogue had already started:

*by completing the form first you feel as if… and the doctor acknowledging that they’ve read it, I think that you’re able to make probably more effective use of the time because the dialogue has already begun. (Patient 38, Feasibility Study)*

These patients felt that completing the form enabled time to be used more effectively in the consultation. Some patients felt that because the GP knew what their problems were in advance, this helped speed the consultation up and save time for the GP. Other patients felt that the consultation was not necessarily shorter, but the time was used more efficiently.

GPs felt that questions about mental health and life support were often reached more quickly when these were completed in the form.

*[It was efficient because] you weren’t having to ask those questions about, how is life, have you got enough support, how’s your mental health […] sometimes when you’re asking patients about those things they’ll go into a huge 10-minute monologue about how they’re struggling, and actually [laughs] you’ve kind of got that little precis of how they’re managing. It’s quite efficient I think. (GP 5, Intervention development study round 3)*

The GP felt that, without the form, she would have had to ask her patients more questions about the impact of their conditions on their lives and their responses would have been much lengthier than was shown on the form.

The context of the form being accurately completed is key to the effective use of time. There were exceptions to the form saving time when patients with ongoing issues may have flagged anxiety or chronic pain on the form, but that wasn’t necessarily the issue that they wanted to talk about that day. In these cases, if the GP raised the issue, then this took more time.

*CMOC 5. GP address what is important to patients-> time used more effectively*

When patients complete the form accurately, and this accurate colour-coded report is shared with the GP (C), consultations time can be used more effectively (O) because GPs can spend the consultation time addressing what is important to the patient (M). ( Box 5)

**Box 5. CMOC 5 diagram**

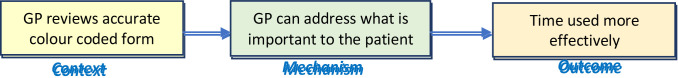

The form enabled GPs to quickly focus on what mattered to the patient, and this meant time was used more effectively. This applied to patients with very straightforward problems as well as complex problems because the GP could focus on that straightforward problem without concerning themselves about a hidden agenda. One patient who was attending because of rectal incontinence and mucus felt the form was useful because it asked about her anxiety and health concerns. The patient was, in fact, not particularly anxious about the problem, but wanted to resolve the symptoms.

*we ruled out are you worried by what you are there for? No, I wasn’t, I was just trying to resolve an embarrassing situation, and that question was kind of covered in a way. Is it causing me a mental issue? No, it’s not. There were certain things there that were ruled out because I answered it honestly. (Patient 34, Feasibility Study)*

Even though this patient did not have any health concerns or anxiety about the problem, she felt it was useful to be asked the question, as she was aware that the GP might have anticipated her having anxiety or concerns about her problem. She pointed out that her accurate responses to the questions were important in making this work.

When complex or multiple issues were raised, being able to focus on what was important to the patient also meant that time could be used more efficiently:

*I think it worked well in terms of sort of getting the relevant information and sort of getting to the heart of the issue earlier on that you probably would have done otherwise in the consultation I think*. *(GP 7, Feasibility Study)*

This GP had anticipated, on doing the training, that there would not be time to deal with the extra issues raised in consultations as a result of the form, but instead found that she was able to “get to the heart of the issue” in terms of what was important to the patient earlier in the consultation and therefore use time more efficiently.

There were some exceptions to this, where the form did not help the GP to focus on the patient priorities. This was when the patient had ongoing chronic problems and completed the detail on the form based on those chronic problems but they did not want to discuss those problems on that day.

*CMOC 6. GP prepare in advance-> time used more effectively*

When patients with a new problem or a problem that requires reference to their medical notes, the BNF formulary or referral/funding rules complete the form accurately, and this accurate colour-coded report is shared with the GP (C), consultations can be more time efficient (O) because the GP can prepare for the consultation in advance (M) ( Box 6)

**Box 6. CMOC 6 diagram**

New problem, or problem requires info from BNF/patient history/referral rules (i.e preparation) and …

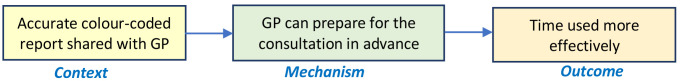

Some patients felt that the GPs were more prepared for the consultation and this meant consultation time was used more effectively. This was particularly when a patient had a problem that required the GP to refer to their medical record, the BNF or funding/referral rules.

*I felt confident that the GP had read the information and was prepared for what I wanted to talk to her about and […] I felt that she had already looked up some test results that I had had previously and was able to comment on those straightaway. (Patient 12, intervention development study, round 3)*

This patient felt that, by reading her form and understanding why she was attending (fatigue, headache and joint pain) the GP was able to look up previous test results, which made the consultation more efficient. GPs also noted that there were times when they were able to be better prepared for the patient by looking up relevant information in advance.

*if you know what they’re asking, particularly those more complicated things around funding rules [patient wanted fertility checks], or those kinds of things, you can look them up beforehand, which is really useful. Normally, I’d have been looking it up afterwards and then saying, ‘Actually, we need to this first,’ or, ‘We need to do that first.’ So, it is really useful for those more complicated things where the rules change all the time, but it’s difficult to remember. (GP 2, Feasibility Study)*
*I was able to prepare. And, looking through their medical records to see any history that I could relate to what they were talking about, whether it was a new. So it allowed me to almost have a mini-plan depending on what the patients would tell me. (GP 4, Feasibility Study)*

GP 2 explained that, knowing the patient was going to request a fertility check, she was able to look up the rules on this in advance, in the context of how the patient’s life was affected, where previously the GP would have had to look up these things afterwards.

**Outcome: Wider range of support offered tailored to the patient’s needs**

*CMOC 7. GPs address what is important to the patient->wider range of support offered tailored to the patient needs*

When patients complete the form accurately, and this accurate colour-coded report is shared with the GP (C), the GP can offer a wider range of support which is tailored to the patient needs (O) because GPs can spend the consultation time addressing what is important to the patient (M). ( Box 7)

**Box 7. CMOC 7 diagram**

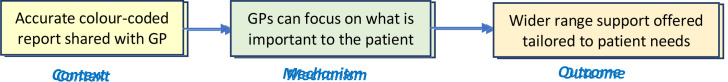

Some patients felt the form enabled the GP to quickly focus on what was important to them, enabling them to deal with multiple issues, where previously there may have been only time for one.

*I think I raised a lot of things on the form. The talk with the doctor was pretty much jam packed. There was no waffling or dallying about. That’s what I mean; it was just very productive because there were a few things on the form. (Patient 44, Feasibility Study)*

This patient went on to describe a targeted conversation, focussed on her needs which resulted in a referral to physiotherapy.

Some GPs also though the form often enabled them to quickly focus on what was important to patients and this meant that they could offer the patient more support, either within the consultation, by a treatment plan, referral or signposting.

One GP explained how this was particularly useful for a patient with an anxiety or a health concern, because the GPs priority is often to focus on a diagnosis and the immediate safety of the patient. However the patient’s priority may be to deal with the anxiety, and this can often be done quickly without compromising the diagnosis or patient safety. Some GPs also felt that they offered a broader, more holistic range of support to patients, because they were more aware of the impacts that their problems were having on their life.

*say for example someone has got pain, normally you’d be focussing on their pain and talking about painkillers, but you wouldn’t have acknowledged, ‘Actually I can see you’re really struggling with this pain, I would like to support you.’ So that was the kind of thing that I was saying at the beginning of the consultation, you’d say that and then you’d say, ‘Well tell me about the pain,’ and go through the normal management of their pain, but then you would say, ‘Well actually let’s think about support we can give you,’ be that mental health support or social prescribing, or follow up consultation. Yes, I think it was good. (GP5, Intervention development study, round 3)*

This GP felt that whereas previously she would have focussed on pain and medication, she now focussed on other support that could be offered. The patient in question, who had been consulting with back pain, also agreed with this assessment:

*You’re not [normally] asked that when you go to the doctors. They don’t say, ‘how does this make you feel?’ It’s kind of, ‘alright here you go’ or ‘we’ll see you in a couple of weeks’. I think that made a massive difference because now I’ve got more help with how I’m actually feeling in myself […] just by simply clicking on a link and answering questions. It has really benefitted me. I would have gone to my appointment, had my back checked over and walked out but now I’ve had several things for me that have really benefited me. (Patient 9, Intervention development study round 3, patient of GP 5 quoted above)*

This patient felt that because she was explicitly asked about the impact of her back pain on her life the GP was able to address those impacts in terms of offering more support.

*CMOC 8. Written record emphasises importance->wider range of support offered tailored to the patient’s needs*

When patients who feel their problems have not been previously addressed or taken seriously complete the form accurately, and this accurate colour-coded report is shared with the GP (C), a wider range of support is offered tailored to the patient needs(O) because the written record emphasises the importance to the GP (M). ( Box 8)

**Box 8. CMOC 8 diagram**

Patient who feels their problems have not been addressed before and …

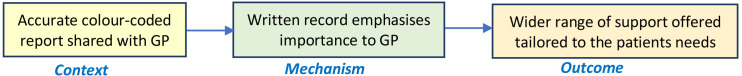

Patients felt the written record was important, not only because it was a reminder for GP so that their GP could raise the issue before the patient did (CMOC3), but also because the written record emphasised the important to the GP and may therefore have prompted action:

*And also, when you’re writing it all down, all your symptoms, you feel more in control, because you’re actually writing it down at the end of the day, rather than going into the doctors and saying it, you’re writing it down. The action of writing it all down makes it more real to the doctor and to you because you’ve written it down there’s a record there. (Patient 28, Feasibility Study)*
*I felt like they almost had a different tone with me [because of the pre-consultation form] Well more than normal because a lot of the stuff you can’t really see it on me. It’s not that they disbelieve me normally but it’s just that I suppose they are being accountable aren’t they because it’s in writing. It’s like this needs to be dealt with or this needs to be talked about. (Patient 44, Feasibility Study)*

For these patients, the written record was more real to GPs than a verbal report. They felt the GP was more accountable because of the written record and they were more likely to be taken seriously.

Some patients believed that action was taken because GPs took the written record seriously. Patient 2 believed the pre-consultation form helped move her consultation forward, and contributed to the GPs decision to refer her to a physiotherapist. When asked how she though the form made a difference she said:

*because I think she could see the words, and I think sometimes words can have a bit more impact written down than sitting face to face. (Patient 2, Intervention Development Study, round 1)*

Patient 2 believed that having the written record emphasised the importance for the GP, increased her accountability and prompted her to make a physiotherapy referral.

GPs did not raise this in earlier interview rounds. Because patients raised it, some GPs in later interviews were explicitly asked, and most disagreed with the patient’s assessment:

*Interesting they feel that. I don’t think the doctor feels like it’s more real. [laugh] (GP 3, Feasibility Study, GP of patient 28 quoted above)*

One GP did agree that he might take the written record more seriously than the verbal account. This GP said that he felt many of his patients often exaggerated their symptoms.

*I would probably say I thought about that less [patient is exaggerating] when I was reading the reports. Maybe them putting it down on paper gives it some kind of more… more believable or more legitimate […] it's an extra step there that takes an extra bit of thought for the patient and maybe that extra thought is what makes it more legitimate, more real. (GP 9, Feasibility Study, GP of patient 44 quoted above)*

This GP felt that the extra step of having to physically write the symptoms down may cause the patient to reflect more and be less likely to exaggerate, so he would take what was written more seriously than what was spoken. GPs acknowledged that the form sometimes highlighted the number of times a patient had consulted about a problem before, and this sometimes made them take the patient’s problem more seriously:

*one thing I did think about this form which is actually in blue was quite useful to know, was that she’d said she’d had nine episodes of depression in 16 months and that was quite useful to know. I perhaps might not have known how frequently she was feeling she was becoming unwell and not functioning […] and not been able to work for a year is quite a big thing isn’t it really. That’s kind of a big red flag if they’re not getting back to work really. So yes, that was quite a useful form especially because I didn’t know this person very well so that was, you know, I do now and yes I’ve had quite a few more consultations subsequently with this person so. (GP 3, Feasibility Study)*

As this GP explained the patient’s multiple depressive episodes was new information for her. However, the patient may have thought that, as this should have been available from her medical record, the GP was taking it more seriously because it was in writing. Another GP said that if the top of the form indicated that the patient had consulted about this problem multiple times, it made her focus more on making active progress towards a resolution within that consultation, which may have created a similar impression among patients.

**Outcome: Patient more satisfied with the consultation**

*CMOC 9. GP Listening and acknowledging->patient more satisfied*

When GPs read the form, let the patient know they have read it, reflect the problems back to the patient and then listen carefully listening carefully (C) patients are more satisfied with the consultation (O) because they feel listened to and taken seriously (M) (particularly when they feel they have not been listened to or taken seriously in the past) ( Box 9)

**Box 9. CMOC 9 diagram**

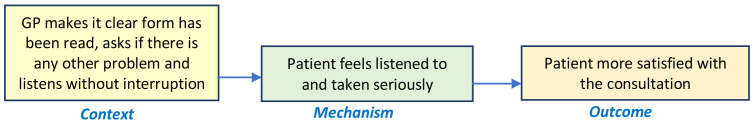

A lot of patients said they felt listened to and taken seriously when GPs acknowledged that they had read the form. This was a key driver of patient satisfaction. Patients who completed the form really appreciated the GP acknowledging that they had read the form immediately. Because GPs were not used to this way of opening a consultation it initially felt awkward to some of them; they were concerned it might interfere with the consultation and might seem like they were listening less than usual. However, most patients thought the opposite – they felt more listened to than usual.

*R: She did listen really well. Yeah, no, I really felt listened to and that does definitely not always happen, so I did. I did come off the phone and I said to my husband, I just said, ‘That was just so good. I actually felt really listened to’, (Patient 23, Feasibility Study)*
*I: You feel the questionnaire was a part of that?*
*R: Yeah, I do. I do.*

This patient attributed the GP listening well to her completing the form in advance. Some patients said it was hard to tell if this was as a result of completing the questionnaire, particularly if it was their first time seeing that GP. One patient who tended to see the same GP explained that she already held this GP in high regard, yet felt more listened to than usual:

*I’ve seen her [the GP] previously a couple of times before this questionnaire. When I received the link, I’d seen her two times prior. It was a completely different experience. I was definitely listened to a lot more. Don’t get me wrong, she’s a wonderful doctor and she listens anyway but this time I felt more listened to. (Patient 9, intervention development round 3, patient of GP 6 quoted below)*

In many cases, the GPs seemed to underestimate the impact of them acknowledging they had read the questionnaire and then listening had in terms of patient satisfaction. When this patient’s GP was asked about whether she thought she listened more she said:

*I don’t think I was listening more than I would have done, but that’s probably just acknowledging more, and actually actively talking back to them what they’ve said. I think just that summary of saying, ‘From what you’ve written I can see that x, y, and z. That’s how we train our students to reflect back to patients, but perhaps I don’t do that enough and it’s making sure that you do that, which is quite nice. (GP 6, intervention development round 3, GP of patient 9 quoted below)*

The GPs explained that she was not listening any more than usual, but that the process of reading the pre-consultation form and reflecting it back to patients probably gave her patients the impression she was listening more. As the GP pointed out, this is not a new technique; these are core consultation skills which are taught to medical students, but under the pressures of a normal daily surgery it is easy to forget the importance of this, and the COAC intervention provided a framework for doing it early in the consultation so the patient felt listened to and validated.

*CMOC 10. GP gauging patient mood and showing empathy->patient more satisfied*

When patients who are feeling overwhelmed or down about their health problems complete the form accurately and GPs read the form patients (C) are more satisfied (O) with the consultation because the GP can gauge the patient’s mood early and show appropriate empathy (M). ( Box 10)

**Box 10. CMOC 10 diagram**

Patient feeling overwhelmed or down about their health problems and …

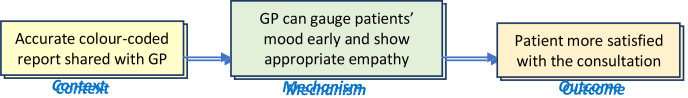

Some patients were satisfied with their consultation partly because the GP treated them with empathy and kindness. These were patient who were feeling low or overwhelmed, and some of these felt the form being shared with the GP was what prompted the GPs manner:

*her knowing it already helped... helped me, especially because she knew I was feeling quite stressed and low and anxious over everything. And knowing that already, she could be a little bit more gentle with me, if you like...a bit more mindful of that fact that I was feeling quite low, if you like […] it was actually quite refreshing to have someone be a bit sympathetic. [Patient 7, intervention development study, round 2)*

This patient felt the GP was more “gentle” with her, because she knew from reading the form how the patient was feeling. This made the patient more satisfied with the consultation, referring to the experience as “refreshing”.

*CMOC 11. Issued addressed and more efficient and supportive consultations->patient more satisfied*

When all their issues are picked up, when the consultation seems efficient and when a wider range of support is offered (Outcomes -> Contexts), patients are more satisfied with the consultation (O), because their needs and expectations have been met (M). ( Box 11)

**Box 11. CMOC 11 diagram**

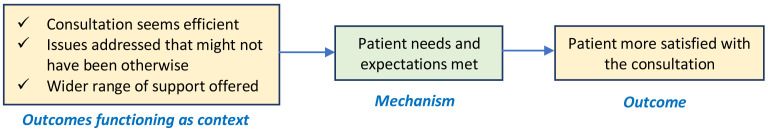

The majority of patients interviewed felt more satisfied with the consultation than usual. Although this was partly because they felt listened to and taken seriously (CMOC 9), patients also felt more satisfied when all their issues were picked up on and they were offered support for them. One patient had completed the form describing headaches, low mood, memory loss and other symptoms which she though was related to the menopause. She expressed described her satisfaction with the consultation as follows:

*That really was easy for me [completing the form]. It was fine. There was nothing that I would have changed. And obviously I got what I wanted at the end. I knew what was wrong. I knew how it should be dealt with, and I got my end result. So, everyone’s a winner really. (Patient 29, Feasibility)*

There was evidence from the interview that the efficiency of the consultation partly led to this sense of satisfaction that the patient described:

*I felt like she’d read everything I put, and then it was just really easy and quick. (Patient 29, Feasibility)*

The patient described it as “easy and quick” because the GP had read the information in advance. The patient also felt that all her issues were addressed.

*I felt like it was, I said because I had the time and space, I said everything that I wanted to say. That there was nothing that I just thought, shit, I should have said that I should have said this, because I’d already said it. (Patient 29, Feasibility)*

Finally the patient felt that the GP was able to provide more support because of the form:

*She read it and made a sensible decision as to how to move forward. (Patient 29, Feasibility)*

In the quote above, patients 29 explains that the GP was not only efficient, and uncovered her concerns, but also provided her with support and a plan on how to “move forward”. This patient therefore provides evidence that these three outcomes led to her satisfaction. The consultation was a telephone consultation and she felt that the pre-consultation form worked particularly well for this.

**Outcome: Improved patient wellbeing**

*CMOC 12. Wider range of tailored support offered->improved wellbeing*

When GPs offer a wider range of support tailored to the needs of the patient (Outcomes -> Contexts), the patient’s wellbeing, health, knowledge or empowerment can improve (O) because they are provided with the means to address their needs (M) ( Box 12)

**Box 12. CMOC 12 diagram**

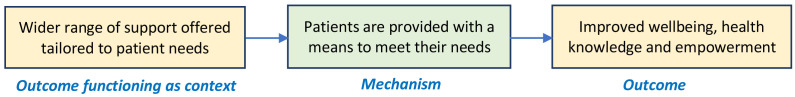

Some patients described how being offered more support improved their well-being, health knowledge and empowerment. Improved wellbeing, health knowledge and empowerment is the most distal outcome shown in the revised programme theory, as often patients have to go through with the treatment plan before their health improves so there is less evidence for this outcome than others. However, there is still some evidence in a small number of patients.

One patient who put “follow-up” as her reason for appointment was there to discuss indigestion and chest pain. She had previously been prescribed Gaviscon, and had a follow-up consultation to check the problem was resolved. Because she was specifically asked on the form, she disclosed that she was struggling to lose weight, and would like some counselling support. The patient described her outcomes:

*Well I was really pleased because, as I say, he referred me, so I’m going to join Weight Watchers, and also I’ve booked an assessment to maybe get some counselling. And also he reassured me about the indigestion. So I felt that it was a really useful conversation [...] I think it [the questionnaire] definitely did help, because I probably wouldn’t have mentioned the other things, we’d have just talked about the indigestion and that would have been it. (Patient 41, Feasibility Study)*

The patient said she would not have raised the other issues had she not been asked in the form, so that additional support she received with lifestyle and mood was as a direct result of completing the form. This patient’s GP was also interviewed and mentioned this patient:

*you’ve gotta remember this is also kind of like not face to face as well so this is where this actually proved quite helpful because […] you know to broach someone’s weight is a bit more difficult over the phone may be than face to face […] And so a five minute telephone call could have very easily been well ‘[how] is your indigestion’, ‘oh it’s a bit better with the over counter stuff’, ‘right fine’. But this gave the option to explore, rather than terminating the call quite quickly, to actually really look into what other things she thinks would be helpful and hence the discussion about her weight which she made the focus of the consultation a lot more. [laugh] you know it’s made the consultation longer but the patient got something more out of it as a consequence. (GP 8, Feasibility Study)*

The GP agreed that the problem probably would not have been raised had the patient not put it on the form. He pointed out that the consultation was not more time-efficient, and in fact took longer, but was more valuable to the patient.

Another patient, who was very positive about the contribution of the pre-consultation form to her consultation outcome was asked how she thought her outcome was improved through completing the form:

*I think an example is that lots of other antidepressants lead to weight gain quite a lot, and in the past a lot of doctors have said that it was just my hormones and things like that. But being able to write down on the form that I wasn't happy with the weight gain, I was able to raise that. I probably wouldn't have raised that before if it was face-to-face, I would have felt they would say something similar about my age, and this, that, and the other […] So, I changed my medication, and I also mentioned on the form – I was able to do so with the multiple boxes – that I had trouble sleeping. So that was taken into consideration, and now I sleep much better, which means I'm generally a happier person! [..] So, I think yeah, it’s had a knock-on effect definitely, because I feel I've had a tailored outcome instead of if I just walked in and tell him I'm struggling with my mental health, he may have just put me on the first medication, the most common one. Even though that's not always the best individually; I was able to have a more tailored consultation. (Patient 47, feasibility Study)*

This patient strongly felt completion of the form led to improved sleep and changed medication, which she hoped would help with weight loss. The patient wrote on her form that she was unhappy with the side-effects of her medication, that she had raised this with multiple GPs and felt that poor continuity of care was hampering her progress. The patient felt that she would not have been able to explain this so succinctly verbally, and that by being able to read her history and concerns on a single form, the GP took action which resulted in immediate improvements to her well-being. 

*CMOC 13. Listening and acknowledging->improved wellbeing*

When GPs read the form, let the patient know they have read it and reflect the problems back to the patient before listening without interruption (C), this can improve the patient’s wellbeing, health knowledge and sense of support (O) because they feel listened to and taken seriously (M) (which is therapeutic in itself). ( Box 13)

**Box 13. CMOC 13 diagram**

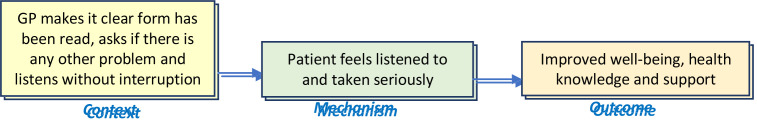

Some patients felt that being listened to and taken seriously was therapeutic in itself, and improved their well-being, often their mood. One patient explained:

*I actually felt really listened to’, because quite often, especially with **(disease**), I can feel completely on my own and I’m having to manage my illness on my own. I said, ‘I actually don’t feel on my own anymore. I actually feel like a doctor listened to me and I’ve got someone working with me in my illness and I’m not on my own anymore’. It made a huge difference, a really big difference. (Patient 23, Feasibility Study)*
*I was more reassured because I felt like I was actually being taken seriously and listened to. (Patient 29, Feasibility Study)*

Patient 23 explained that being listened to made a “huge difference” not just in terms of her satisfaction with the consultation, but with her sense of support going forward. She no longer feels on her own with her illness. Patient 29 described herself as feeling reassured because she was listened to. As with the other CMOC related to improved well-being, there was less evidence for this than the other outcomes.

**Outcome: Patient confidence in seeking healthcare**

*CMOC 14. GP Listening and acknowledging->patient confident in seeking healthcare*

When patients feel their problems have not previously been addressed or taken seriously, and GPs read the form, let the patient know they have read it and reflect the problems back to the patient and then listen carefully (C), patients are more confident in seeking healthcare in the future (O) because they feel they have been listened to and taken seriously this time (M). ( Box 14)

**Box 14. CMOC 14 diagram**

Patient who feels their problems have not been addressed before and …

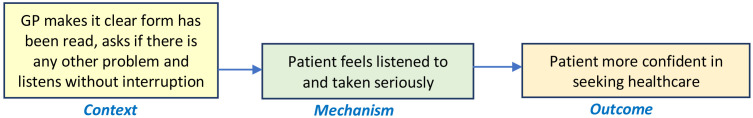

Some patients felt that, because they felt more listened to, they were more confident in seeking healthcare from their GP in the future. This was particularly the case for patients who did not feel they had been well listened to before. One patient explained some previous experiences of GP appointments:

*Because of the nature of how difficult it is to see the same doctor each time, I find it very difficult for them to have the time to listen and really understand what’s going on, especially because my needs are sometimes quite complex. So yeah, I do find it very difficult. (Patient 23, Feasibility Study)*

This patient went on to explain that the GP in the COAC appointment had made it clear they read the form and then really listened to her:

*it enabled me to feel like I can talk about things and say exactly what was going on, which just opens up the patient/doctor relationship more. That means that I’m more likely to see the doctor again next time if I have concerns, which I think, certainly from my point of view, makes it a lot safer. (Patient 23, Feasibility Study)*

As a result of the GP making it clear they read the form, this patient felt listened to and taken seriously and more confident in seeking healthcare in the future.

## 4 Discussion

### 4.1 Main findings

This paper reports on the person-based development of a pre-consultation form and on a realist evaluation of this which was embedded within a feasibility study. The person-based development was highly successful. Numerous improvements were made and GPs and patients agreed the final version was much improved on the initial version. In the feasibility study the pre-consultation questionnaire was tested in a single intervention with a summary report. Through the embedded realist evaluation, we found that these were useful for different types of patient. The pre-consultation form is most useful for patients with complex problems, mental health issues, health concerns, a concern they find it difficult to voice, or who find consultations nerve-racking. It was also useful for patients who sometimes feel that the GP doesn’t listen to them or understand their problems. It was less useful when patients who completed it had a quick problem, or when they had underlying chronic problems that were unrelated to their consultation.

We identified six possible outcomes of the pre-consultation form which is captured in our finalised programme theory. The previous programme theory ( Figure 3) had included reduction in re-consultation rates as an outcome. However, interviews did not show any evidence that this is a likely outcome. The two outcomes with the most qualitative evidence were: 1) issues discussed which might not have been discussed otherwise and 2) a wider range of tailored support offered to patients. There was also evidence of more time efficient consultations, greater patient satisfaction, increased confidence in seeking healthcare and improved well-being health knowledge and support.

### 4.2 Strengths and limitations

The person-based approach was an effective method of developing the pre-consultation and consultation summary reports. The PPI group were actively engaged and an important part of designing of the intervention. Practices were effectively re-engaged following the COVID-19 related study pause. The REDCap system used for data collection enabled patient-reported data to be collected accurately with no data entry error. An effective collaboration was developed with the GP Federation for BNSSG CCG (One Care) who worked were able to publish the required EMIS resources to the GP practices. Pre-consultation form completion rates increased from 15%-17% in the first two practices to 50% in the final practice.

The realist evaluation is a well-established theory-based approach for making sense of why, when and for whom context sensitive outcomes occur in complex interventions, such as the pre-consultation form. We had a wealth of data across 63 interviews and used rigorous methods to analyse this data within a 3-person team. We were unable to interview non-responders, so we do not know about the type of person who did not respond to the questionnaire. Furthermore, the realist evaluation was restricted to people who agreed to an interview, thereby forming a self-selecting group who either displayed a level of altruism or were more likely to be engaged by the intervention, and this may have affected the findings.

### 4.3 Comparison with literature

There are varied levels of existing quantitative evidence for the outcomes, mechanisms and contexts identified in our programme theory. Our interviews identified that the questionnaires uncovered issues that might not otherwise have been discussed. This included when the patient had a sensitive problem that they might be reluctant to disclose. Previous studies have found that electronic questionnaires are well suited to exploring issues of a complex, personal or sensitive nature, as patients may be more willing raise sensitive problems on a questionnaire than verbally ^43– 45^. A 2020 systematic review showed that use of pre-consultation electronic forms helped patient to disclose more psychosocial and quality of life issues ^46^. The form also helped to pick up on low mood and anxiety. Another 2020 systematic review found that primary care patients were often reluctant to disclose emotional concerns that included sub-clinical low mood, stress and/or anxiety, and low mood, stress and/or anxiety attributable to difficult life circumstances ^47^.

Making consultations more efficient was an important outcome perceived by patients and GPs. Sometimes this involved speeding the consultation up, and sometime using time to better effect. GP consultations are in the UK are 10.9 minutes long on average. GPs in England spend longer with patients who have more conditions, but, at all multimorbidity levels, those in deprived areas have less time per GP consultation ^48^. This intervention could help free up GP time to use with the patients who most need it.

The intervention also seemed to improve patient satisfaction. Good physician–patient communication is central to good patient experience, and a major driver of overall patient assessments of primary care ^49^. The pre-consultation form helped communication on a number of levels: by helping the patient raise problems, helping the GP prepare and focus on what the patient needs and helping the patient feel that they were listened to and taken seriously. Patients were more likely to raise problems about low mood and health concerns if invited to on the form. Previous research has also shown that patients with mental health needs may fail to access support for these needs if they believe they fall outside the scope of primary care in the absence of any physical symptoms ^50, 51^. A specific invitation to share their needs may help with this barrier to access ^45^. Previous cohort studies have also shown high patient satisfaction following completion of a pre-consultation questionnaire, however studies with a control are have normally shown no difference in patient satisfaction ^46^.

A number of patients commented that the GP reading their questionnaire, letting them know they had read it and then listening made them feel more listened to and taken seriously than they had done before. Previous qualitative studies have identified feeling “listened to” as hugely important element of the consultation ^52^. Jagosh *et al.* identified that listening was (a) an essential component of clinical data gathering and diagnosis; (b) a healing and therapeutic agent; and (c) as a means of fostering and strengthening the doctor-patient relationship ^13^. In our programme theory, we similarly identify that listening is a mechanism for improved patient satisfaction, increased patient confidence in seeking healthcare, and improved wellbeing, as listening is therapeutic in itself.

Research shows that, alongside the switch to telephone and video consultation in March 2020, there was a concurrent reduction in diagnoses in primary care ^53^ and a reduction in the number of routine monitoring tasks by GPs, including health promotion ^54^. Our study provides qualitative evidence that the COAC pre-consultation form might help increase diagnoses and monitoring in telephone consultations by helping patients disclose more concerns and focussing GPs on what matters to the patient.

## 4.4 Conclusions

The pre-consultation form was developed and tested using rigorous methods and has been demonstrated to be valuable for both patients and GPs. It is most useful for patients with complex problems, mental health issues, health concerns, a concern they find it difficult to voice, or who find consultations nerve-racking. For these patients, it can reveal issues which might not have been discussed otherwise and lead to a wider range of tailored support offered to patients. It may also make consultations more time efficient and lead to greater patient satisfaction and well-being. However, the administrators implementing the process required too much support from the study research teams for it to be practical to roll-out using the current technological platform. This paper provides information on how to develop such a technology and describes why it works, for what patients under what circumstances for the benefit of future developers of similar technologies.

## Data availability

### Underlying data

Researchers can apply for this data via a form on the repository:

University of Bristol: COAC Study Qualitative Dataset, https://doi.org/10.5523/bris.1ljvagu1sigje2duqj3ube527y (restricted access) ^55^.

This project contains the qualitative data transcripts for the COAC Feasibility Study, where participants agreed that these could be shared with bona fide researchers outside the Bristol research team. Information about each transcript is listed below, as follows:

Transcript ID: The name of the transcript in the folder. The name consists of:

* a participant identifier

* the type of participant (patient, clinician or administrator)

* the site (1 to 4 – this was not reported in the paper for reasons of anonymity)

* The date of the interview

Participant identifier used in papers: This is the identifier used in this paper.

The folder also contains the consent form. All patients in this study consented to point 7 in this form: "I understand that after the study my anonymised data will be made available to bona fide researchers for future research studies, and it will not be possible to identify me from these data. If I agree to this, my data will be held for twenty years."

This dataset has an access level *Restricted*, which means it is not available via direct download but must be requested. Research participants did not give explicit consent to share this data as open data but agreed that it should be made available to approved bona fide researchers only, after their host institution has signed a Data Access Agreement. In order to request access to this data please complete the data request form available from the link above. We will consider any application from any organisation where an established research governance process is in place.

Data are available under the terms of a non-Commercial Government Licence for public sector information.

### Extended data

University of Bristol: COAC Study Extended Dataset, https://doi.org/10.5523/bris.386dsq2e4iii225ms7du8pd5jq ^56^

This project contains the following extended data:

- COAC-pre-consultationForm.doc
  This file contains screenshots of the pre-consultation form which patients responded to in the COAC Study.
- COACStudy-pre-consultationform-TableOfChanges.doc
  This file contains a detailed table of changes made to the pre-consultation form in the COAC Intervention Study. Patients who are quoted in this table all consented to the first six points in the consent form included in this folder.
- COACStudy-SummaryReport-TableOfChanges.doc
  This file contains a detailed table of changes made to the summary report in the COAC Intervention Study. Patients who are quoted in this table all consented to the first six points in the consent form included in this folder.
- COACStudy-TopicGuides.doc
  This file contains the interview topics guides for the COAC Study.
- PatientConsent-Interviewsv1.3.doc
  This is the patient consent form used for the COAC Study
- PatientInfoInterviewStudy2v1.4.doc
  This is the patient information leaflet given to patients interviewed for the COAC Study
- COREQ checklist - pre-consultation form
  This is a checklist for the COREQ reporting guidelines which demonstrates how they were following in collecting and analysing data about the pre-consultation form
- COREQ checklist – summary report
  This is a checklist for the COREQ reporting guidelines which demonstrates how they were following in collecting and analysing data about the summary report

### Reporting guidelines

University of Bristol: COREQ checklist ^57^ for COAC Study, https://doi.org/10.5523/bris.386dsq2e4iii225ms7du8pd5jq ^56^.

The realist evaluation also followed the RAMESES II reporting standards for realist evaluations ^58^.

Data are available under the terms of the Creative Commons Attribution 4.0 International license (CC-BY 4.0).
