# Supplementary material for: The person-based development and realist evaluation of a pre-consultation form for GP consultations
Source: NIHR Open Res. 2022 Jul 29;2:19. Originally published 2022 Feb 17. [Version 2] doi: 10.3310/nihropenres.13249.2 (PMC7614962; doi:10.3310/nihropenres.13249.2)
Supplement: Peer review history

# Reviewer report: Reviewer response for version 2

**DOI:** 10.3310/nihropenres.14409.r28730

**Published:** 2022-08-19

**Authors:** Abi Eccles

**Affiliations:** 1 Unit of Academic Primary Care, Warwick Medical School, University of Warwick, Coventry, UK

Authors have done a great job at responding to my comments and making relevant changes. The examples of patients feeling they can discuss 'hidden' issues are particularly useful demonstrating an important advantage of this tool. 

 Thank you for citing my paper on online triage. However, I still think readers would benefit from further explanation about how this tool differs to online triage. You have explained that the tool could be used to a range of concerns, but this does not distinguish it from online triage where patients are not limited in what they can write. Some discussion about how this tool is more sophisticated, perhaps how was developed, how it actively encourages patients to highlight a broader range of problems, or content is directly used using the consultation would be useful.

Is the work clearly and accurately presented and does it cite the current literature?

Partly

If applicable, is the statistical analysis and its interpretation appropriate?

Not applicable

Are all the source data underlying the results available to ensure full reproducibility?

Partly

Is the study design appropriate and is the work technically sound?

Yes

Are the conclusions drawn adequately supported by the results?

Yes

Are sufficient details of methods and analysis provided to allow replication by others?

Partly

Reviewer Expertise:

Qualitative health researcher working on a range of digital access projects.

I confirm that I have read this submission and believe that I have an appropriate level of expertise to confirm that it is of an acceptable scientific standard.

# Reviewer report: Reviewer response for version 1

**DOI:** 10.3310/nihropenres.14365.r28574

**Published:** 2022-05-09

**Authors:** Abi Eccles

**Affiliations:** 1 Unit of Academic Primary Care, Warwick Medical School, University of Warwick, Coventry, UK

Thank you for inviting me to review this paper, which was very interesting to read. This is very comprehensive and rigorous work evaluating the development and feasibility of a consultation tool. The problem and aims were laid out well. The lay summary is very clear and engaging. There was excellent detail and background describing the CMOC process.

 There are some areas that would benefit from further refinement. It would be useful to have some more detail regarding the methods. So, how participants were selected for interviews if there was a sampling strategy. I was also interested to know what happened to the control group. I appreciate that is not the nature of this evaluation, but perhaps a brief note or reference to where it would be reported might be useful

 It is a little unclear whether the REDCap questionnaire was collecting baseline data or the pre consultation tool or both. One of the flow diagrams just says baseline. It might be helpful to clarify this and add a note that it is the pre consultation tool (as well as the baseline data collection) in any diagrams.

 A definition of BNF might be useful for those unfamiliar.

 Some areas that could do we more consideration and discussion include:

- Patients’ hidden concerns or worries - I was not convinced that the tool uncovered these if it is just based on a judgement GPs made. Was there any evidence from what patients said?
- Those patients who opted not to complete the form. What percentage didn’t? Was there any indication why it was not appealing to those individuals? What can be done to engage them? Also, has there been any further consideration to transfer to non-digital (as highlighted by the PPI group).

It would be interesting to know more about how long the form took to complete for patients and whether the pre consultation review added much admin time to GP’s workload.

 This pre-consultation questionnaire has some similarities to the online triage initiatives (AskMyGP econsults). I think it would be useful to refer to these and point out explicitly how this tool differs. There are some similarities in literature describing the impacts of these online triage tools for patients and GPs too, which would be useful to reference.

 There was a few typos:

- Pg 3 – apostrophes missing from “patients reason” (p.3) “patients interests” (p.4).
- I suggest using term co-investigators, rather than co-applicants (as funded) (p.7,8).
- P.8 “assigned a code to it” is a little unclear. Do you mean assigned a code to each change suggested? Could perhaps clarify.
- Inconsistency with randomisation/randomization (p.9).
- Word missing: “Researcher then took informed …” (p.9).
- Letter missing: Figure 14: 'Feeing' instead of 'feeling'.
- Letter missing: 'Though' instead of 'thought' (p.17+24+24+26).

Is the work clearly and accurately presented and does it cite the current literature?

Partly

If applicable, is the statistical analysis and its interpretation appropriate?

Not applicable

Are all the source data underlying the results available to ensure full reproducibility?

Partly

Is the study design appropriate and is the work technically sound?

Yes

Are the conclusions drawn adequately supported by the results?

Yes

Are sufficient details of methods and analysis provided to allow replication by others?

Partly

Reviewer Expertise:

Qualitative health researcher working on a range of digital access projects.

I confirm that I have read this submission and believe that I have an appropriate level of expertise to confirm that it is of an acceptable scientific standard, however I have significant reservations, as outlined above.

# Response

**Published:** 2022-05-14

**Authors:** Mairead Murphy

**Affiliations:** University of Bristol, UK

Dear Abi, Thanks for this useful review. We will certainly make some changes based on this and submit another version. Mairead

# Reviewer report: Reviewer response for version 1

**DOI:** 10.3310/nihropenres.14365.r28554

**Published:** 2022-04-20

**Authors:** Seán Paul Teeling

**Affiliations:** 1 Centre for Person-Centred Practice research, Queen Margaret University, Edinburgh, UK; 2 Health Sciences Centre, School of Nursing Midwifery and Health Systems, University College Dublin (UCD), Dublin, Ireland

Thank you for the opportunity to review this excellent paper. The paper addresses a key issue in health services internationally - the lack of time available to busy clinicians to spend the time they would like to spend with their patients, leading to a consultation that can be about the condition rather than the person. The paper clearly details the iterative refinement of the consultation form through engagement with both patients and the GP practice teams so that the 'customer voice' is captured throughout.  It is clear that this led to the development of a more person-centred consultation process that works for both patients and the practice staff. The paper illustrates important contextual factors and mechanisms within the process of GP patient consults that can be applicable to those seeking to improve both patient and staff experiences of care.

Is the work clearly and accurately presented and does it cite the current literature?

Yes

If applicable, is the statistical analysis and its interpretation appropriate?

Not applicable

Are all the source data underlying the results available to ensure full reproducibility?

Yes

Is the study design appropriate and is the work technically sound?

Yes

Are the conclusions drawn adequately supported by the results?

Yes

Are sufficient details of methods and analysis provided to allow replication by others?

Yes

Reviewer Expertise:

Person-centred care, Person-centredness, Person-centred Cultures, Process Improvement, Quality Improvement, Realist Evaluation.

I confirm that I have read this submission and believe that I have an appropriate level of expertise to confirm that it is of an acceptable scientific standard.

# Response

**Published:** 2022-04-29

**Authors:** Mairead Murphy

**Affiliations:** University of Bristol, UK

Thank you Sean for your kind words and for this helpful review. Mairead
